# Cellular Exposure to Chloroacetanilide Herbicides Induces Distinct Protein Destabilization Profiles

**DOI:** 10.1021/acschembio.3c00338

**Published:** 2023-07-10

**Authors:** Guy M. Quanrud, Ziqi Lyu, Sunil V. Balamurugan, Carolina Canizal, Hoi-Ting Wu, Joseph C. Genereux

**Affiliations:** Department of Chemistry, University of California, Riverside, California 92521, United States

## Abstract

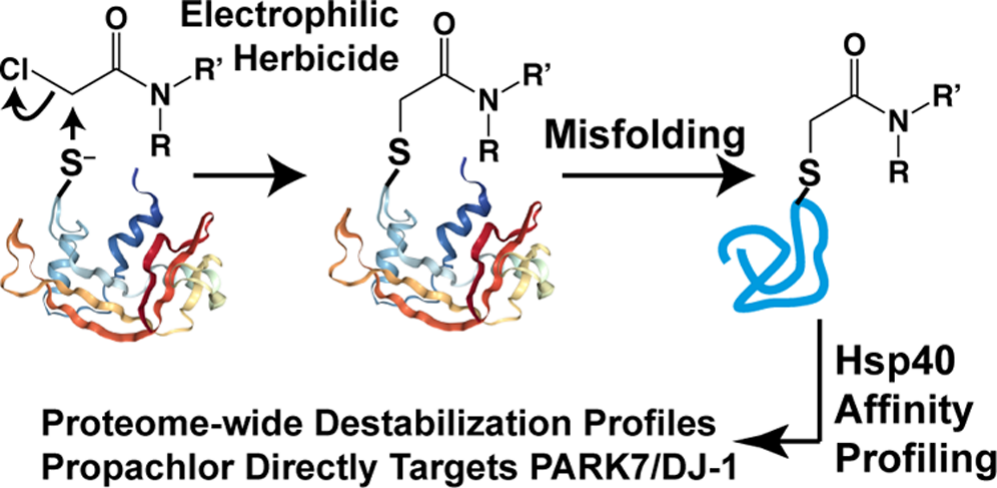

Herbicides in the widely used chloroacetanilide class harbor a potent electrophilic moiety, which can damage proteins through nucleophilic substitution. In general, damaged proteins are subject to misfolding. Accumulation of misfolded proteins compromises cellular integrity by disrupting cellular proteostasis networks, which can further destabilize the cellular proteome. While direct conjugation targets can be discovered through affinity-based protein profiling, there are few approaches to probe how cellular exposure to toxicants impacts the stability of the proteome. We apply a quantitative proteomics methodology to identify chloroacetanilide-destabilized proteins in HEK293T cells based on their binding to the H31Q mutant of the human Hsp40 chaperone DNAJB8. We find that a brief cellular exposure to the chloroacetanilides acetochlor, alachlor, and propachlor induces misfolding of dozens of cellular proteins. These herbicides feature distinct but overlapping profiles of protein destabilization, highly concentrated in proteins with reactive cysteine residues. Consistent with the recent literature from the pharmacology field, reactivity is driven by neither inherent nucleophilic nor electrophilic reactivity but is idiosyncratic. We discover that propachlor induces a general increase in protein aggregation and selectively targets GAPDH and PARK7, leading to a decrease in their cellular activities. Hsp40 affinity profiling identifies a majority of propachlor targets identified by competitive activity-based protein profiling (ABPP), but ABPP can only identify about 10% of protein targets identified by Hsp40 affinity profiling. GAPDH is primarily modified by the direct conjugation of propachlor at a catalytic cysteine residue, leading to global destabilization of the protein. The Hsp40 affinity strategy is an effective technique to profile cellular proteins that are destabilized by cellular toxin exposure. Raw proteomics data is available through the PRIDE Archive at PXD030635.

## Introduction

Reactive environmental toxins are a threat to proteome integrity. Heavy metals damage protein through oxidation or direct binding, while electrophiles form covalent adducts.^1−3^ Damaged proteins can become misfolding-prone, and all proteins, if misfolded, have the potential for aggregation and proteotoxicity.^4^ Misfolded proteins threaten cellular proteostasis, causing proteins that are not directly targeted to misfold and further threatening cellular function.^5−7^ Despite this risk, the challenge of characterizing proteome integrity has limited studies of how environmental exposures threaten the proteome.^3,8−11^

**Figure 1:**
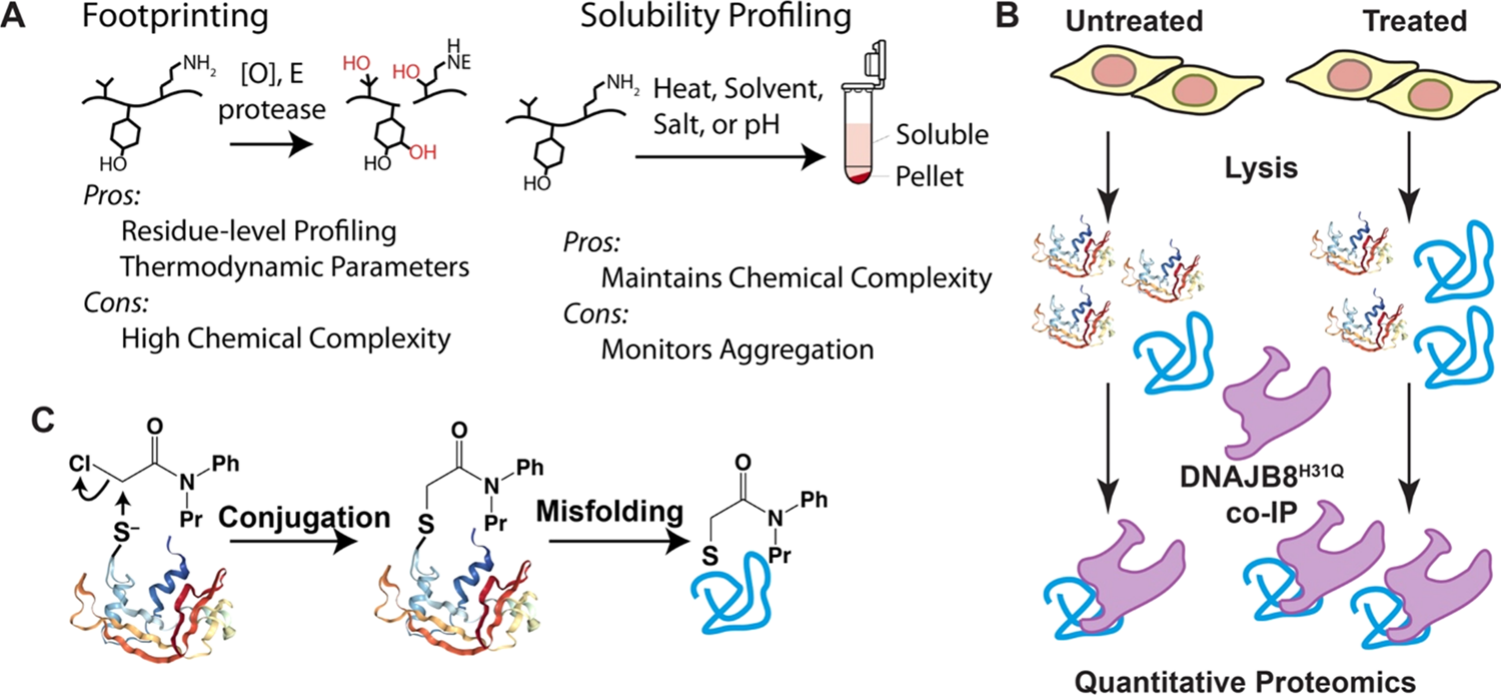
(A) Description of footprinting and solubility profiling approaches for identifying changes in protein conformation and stability. [O] denotes reactive oxygen species, E denotes an electrophile. (B) Description of our assay to identify changes in protein stability based on affinity to the Hsp40 DNAJB8^H31Q^. (C) Propachlor conjugation to cysteine as a mechanism for protein destabilization.

The development of screening techniques to identify the protein targets of small molecules has been transformative for chemical biology, protein science, and the pharmaceutical industry.^12−14^ Activity-based protein profiling (ABPP) can be used to discover the protein targets of a covalent ligand.^15−17^ ABPP can also be used to determine targets of a noncovalent ligand based on its competition with a general covalent probe. Footprinting techniques identify proteins that change conformation or dynamics in response to a small molecule exposure,^18−23^ including toxicants,^24,25^ while solubility-based methods can identify protein targets by profiling changes in protein aggregation susceptibility to temperature, solvent, ionic strength, or pH^26−29^ (Figure 1A). All of these approaches are enabled by advances in mass spectrometry-based quantitative proteomics. Competitive ABPP can only profile proteins that lose activity at a reactive residue, while the other techniques require profiling the entire proteome. Footprinting approaches in particular can substantially increase the chemical complexity of the samples, further challenging the depth of profiling by mass spectrometry.

We previously developed an affinity purification mass spectrometry (AP-MS) approach to profile the misfolded proteome based on its affinity to the human Hsp40 DNAJB8 (Figure 1B).^30^ This assay combines affinity purification of overexpressed ^Flag^DNAJB8^H31Q^ with quantitative proteomics to identify hundreds of coisolating cellular proteins with high reproducibility and statistical confidence.^31,32^ The H31Q mutation blocks the release of misfolded proteins from DNAJB8, making it a thermodynamic sink for its clients. This binding is highly detergent-resistant, allowing immunoprecipitates to be stringently washed. Proteins that are destabilized by a treatment are enriched during affinity purification and hence are more likely to be identified during LC-MS in data-dependent analysis mode.

It is important to note that this approach is not profiling endogenous clients of DNAJB8. First, DNAJB8 is not expressed in HEK293T cells but rather only in the testes.^33,34^ Second, native Hsp40 interactions are often transient and localized, and careful experimental and statistical methods are necessary to ensure that biological clients are being identified.^35^ Indeed, we identify many proteins associating with DNAJB8 that are expressed in other compartments, indicating that recognition is taking place post lysis.^31,32^ This is a benefit of the approach from the perspective that it broadens the range of misfolded cellular proteins that can be profiled.

Herein, we apply our Hps40 affinity platform to identify proteins that are destabilized after cellular exposure to chloroacetanilide herbicides, a widely used herbicide class harboring a highly electrophilic haloacetamide motif (Figure 1C).^36−39^ Although previous work demonstrated that acetochlor targets several hepatic fatty acid binding proteins in mice,^15^ the protein destabilization profile has not been determined, and the target overlap between different chloroacetanilides has not been addressed. We find that although the structurally similar acetochlor, alachlor, and propachlor herbicides generally target proteins with reactive cysteines, the actual targets are highly specific to the herbicides that are used. We also find that propachlor in particular targets Parkinson’s disease-associated proteins GAPDH and PARK7, decreasing their cellular activity.

## Materials and Methods

### Materials

We purchased 1,4-dithiothreitol (DTT), Roche protease inhibitor cocktail w/o EDTA (PIC), HEPES, propachlor, acetochlor, alachlor, Tris(2-carboxyethyl)phosphine hydrochloride (TCEP), sepharose-4B beads, M2 anti-Flag magnetic dynabeads, and the GAPDH activity assay kit (catalog #MAK277) from Sigma-Aldrich. We purchased bovine serum albumin (BSA), Dulbecco’s modified Eagle’s Media (DMEM), Dulbecco’s phosphate-buffered saline (DPBS), 10 cm tissue culture plates, and 6-well tissue culture plates from VWR. We purchased KCl, MgCl_2_, CaCl_2_, Ag(NO_3_)_2_, Na_2_S_2_O_3_, NaCl, Tris–HCl, Triton X-100, sodium deoxycholate, urea, calcium acetate, glycerol, sodium dodecyl sulfate (SDS), poly-d-lysine, and sequencing grade trypsin from Thermo Fisher Scientific. Proteinase K (PK) and trypsin/LysC mix were purchased from Promega. Resazurin sodium salt was purchased from Acros Organic. Dried skim milk was purchased from Walmart. Nanopure water was prepared from a Millipore Milli-Q Laboratory Lab 4 Chassis Reagent Water System. 5 μm and 3 μm Aqua C18 resins were purchased from Phenomenex. 250 μm inner diameter-fused silica columns were purchased from Agilent. 100 μm inner diameter-fused silica columns were obtained from Polymicro. The strong cation-exchange resin was from Partisphere, GE Healthcare. Rapigest was purchased from Aobious (Gloucester, MA). TMT 6-plex isotopic labels were from Pierce. Bradford reagent was purchased from Bio-rad. We purchased 5-Hex-5-ynyl-2-iodoacetamide (IAA-alkyne), Tris(2-carboxyethyl)phosphine hydrochloride (TCEP), copper(II) sulfate (CuSO_4_), Tris[(1-benzyl-1*H*-1,2,3-triazol-4-yl)methyl]amine (TBTA), and Azo biotin-azide from Sigma-Aldrich. The ambient temperature in our laboratory is maintained between 17 and 21 °C.

### Cell Culture and Immunoblotting

HEK293T cells (ATCC) were maintained in DMEM supplemented with glutamine, penicillin, streptomycin, and fetal bovine serum (Seradigm). ^Flag^DNAJB8^H31Q^ plasmid has been reported previously.^31^^Flag^GAPDH in the pCMV3 vector was purchased from Sino Biological. Immunoblots were transferred to nitrocellulose from SDS-PAGE gels using a Bio-Rad Trans-Blot Turbo, stained with ponceau S to image total protein, blocked with 5% dried milk/TBST (10 mM Tris pH 7.4, 150 mM NaCl, 0.1% Tween), washed with TBST, incubated with primary antibody in 5% BSA/TBS with 0.1% NaN_3_, washed with TBST, incubated with a near-IR-conjugated secondary antibody (Li-COR) in 5% dried milk/TBST, washed with TBST, washed with TBS, and imaged on a Li-COR Fc. Mouse monoclonal M2 anti-Flag was purchased from Sigma-Aldrich. Rabbit polyclonal anti-HSPA1A and mouse monoclonal anti-β-actin (7D2C10) were purchased from Proteintech. Mouse monoclonal anticarboxymethyllysine (#318003) was purchased from R&D Systems.

### Hsp40 Affinity Profiling

TMT-AP-MS experiments using ^Flag^DNAJB8^H31Q^ were performed as described previously.^30^ Briefly, six 10 cm plates of HEK293T cells were transfected by the calcium phosphate method with 5 μg of plasmid DNA encoding ^Flag^DNAJB8^H31Q^ in the pFLAG backbone. Plates were treated with 1 mM of the indicated chloroacetanilide at 40–46 h post transfection for 30 min in serum-free media. Cells were harvested by scraping in DPBS. Cells were then lysed in 9 parts of radioimmunoprecipitation assay (RIPA) buffer (150 mM NaCl, 50 mM Tris pH 7.5, 1% Triton X-100, 0.5% sodium deoxycholate, 0.1% SDS) and 1 part of 10× PIC on ice for 30 min. Samples were centrifuged 21,000*g* for 15 min at 4 °C to separate lysate from cell debris. The Bradford assay was used to quantify protein in each lysate. Lysates were incubated with 15 μL of Sepharose-4B beads for 30 min at 4 °C and then centrifuged at 1500*g* for 1 min to pellet beads. Lysate was then separated and then incubated with 15 μL of M2 anti-Flag magnetic beads and rotated overnight at 4 °C. The anti-Flag beads were washed the next day four times with RIPA buffer. Each wash included rotation for 10 min at an ambient temperature. Proteins bound to the anti-Flag beads were eluted by boiling for 5 min at 100 °C in 30 μL of Laemmli concentrate (120 mM Tris pH 6.8, 60% glycerol, 12% SDS, brilliant phenol blue to color). 5 μL of the elutes were saved for silver stain analysis, and the remainder was prepped for mass spectrometry and TMT-labeled from a 6-plex TMT set.^40^

Only MS quality organic solvents were used during sample preparation. Samples were CHCl_3_/MeOH precipitated, resuspended in 1% Rapigest, and diluted in 50 mM HEPES pH 8.0. Rapigest is a powerful ionic detergent that efficiently solubilizes protein precipitates and upon acidification is cleaved to a hydrophilic salt and an insoluble ketone that are readily purified out prior to MS.^41^ Samples were then reduced for 30 min with 5 mM TCEP, alkylated for 30 min in the dark with 10 mM iodoacetamide, digested with 500 ng of trypsin overnight at 600 rpm and 37 °C, labeled with the appropriate TMT tag NHS-ester in 40% acetonitrile (ACN) for 1 h, quenched with ammonium bicarbonate, pooled, acidified by being brought to 5% formic acid, and clarified by centrifugation for 30 min at 21,000*g*. The composition for buffer A is 0.1% formic acid and 5% ACN in water. The composition for buffer B is 0.1% formic acid and 80% ACN in water. The composition for buffer C is 500 mM ammonium acetate in buffer A. MS runs were performed by using a two-dimensional LC/MS/MS setup on an LTQ Orbitrap Velos Pro hybrid mass spectrometer (Thermo) interfaced with an Easy-nLC 1000 (Thermo) according to standard MuDPIT protocols.^42^ For each run, MS/MS spectra were extracted using MSConvert (version 3.0.21144) with peak picking filtering. FragPipe was used to search MS/MS spectra against a Uniprot human proteome database (06/11/2021 release, longest entry for each protein) supplemented with common contaminants and reverse sequence decoys for a total of 40,858 sequences.^43^ MS/MS spectra were also searched against 20,429 select decoys (e.g., albumen, porcine trypsin, contaminants, etc.). FragPipe searches allowed for the static modification of cysteine residues (57.02146 Da, acetylation), variable modifications of herbicide adducts (175.0997 for propachlor adducts and 233.1416 for acetochlor and alachlor adducts), static TMT tagging of N-termini and lysine residues (229.1629 Da), and half-tryptic peptidolysis specificity. We allowed a mass tolerance of 1.25 Da for the precursor ion mass and 20 ppm for the product ion masses. MSFragger (version 3.2) was used to match and filter spectra. Decoy proteins, common contaminants, immunoglobulins, and keratins were filtered from the final protein list. Quantitation in FragPipe was performed by averaging TMT reporter ion intensities for all spectra associated with an individual peptide. Raw proteomics data is available through the PRIDE Archive at PXD030635.

### AP-MS of GAPDH

^Flag^GAPDH AP-MS was performed similarly, except digestion was performed using a two-step LysC/trypsin (Promega) digestion according to the manufacturer’s protocol, and dynamic exclusion was set to 30 s during the LC-MS/MS analysis of peptides. Open search was performed in FragPipe using default settings.^44^ For intact protein MS, beads were washed 4 times with PBS after RIPA washes and eluted overnight in 8.8 M urea in 50 mM Tris pH 8.0. Quantitative immunodepletion was confirmed by immunoblotting, sample purity was confirmed by SDS-PAGE followed by silver stain, and samples were analyzed by LC-ESI-MS on an Agilent 6545 LC/QTOF. Charge state envelopes were deconvoluted using MassHunter Bioconfirm software.

### Statistical Analysis

Statistical analysis was performed as previously described.^30^ Initially, protein-level intensities were normalized to the intensity of bait (DNAJB8) in each TMT channel. We then used a version of the scaled reference approach to combine multiple TMT runs.^30,45^ The bait-normalized integrated TMT reporter ion intensities were averaged for each protein across the three control conditions in each AP-MS run to get a scaling factor. Each bait-normalized protein intensity was then divided by this scaling factor. Storey’s modification of the method of Benjamini and Hochberg was used to convert unadjusted *p*-values to *q*-values (local false discovery rates).^46,47^ Unadjusted *p*-values were ranked in increasing order, and the *q*-value for the *i*th protein was determined from
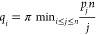
Storey’s modification is performed by determining the overrepresentation of low *p*-values to infer a global false discovery rate and then scaling local false discovery rates accordingly. The π-factor for this scaling was 0.54 for the acetochlor treatment, 0.5 for alachlor treatment, and 0.3 for propachlor treatment.

### Limited Proteolysis and PRM

The limited proteolysis (LiP) procedure was optimized from standard protocols and previous experiments.^22,30^ 1 mg/ml stocks were prepared from 25 mg of lyophilized proteinase K (PK) dissolved in storage buffer (50 mM Tris–HCl, 2 mM calcium acetate, pH 8.0) and stored at −70 °C. The following concentrations of PK were prepared from serial dilutions from the 1 mg/mL aliquot: 0.5, 0.2, 0.1, and 0.05 mg/mL, and added to lysate to yield 1:200, 1:500, 1:1000, and 1:2000 protease:substrate protein ratios (w/w), respectively. 2 μL of PK was added to a 200 μg aliquot of protein lysate and incubated for 1 min at 25.0 °C for each digestion. Samples were then boiled for 5 min to quench PK activity. Three separate digestions were performed for the no PK condition for each lysate sample. Samples were prepared for mass spectrometry and analyzed using LC-MS/MS and parallel reaction monitoring (PRM). Chromatograms and product ions were quantified by Skyline.^48^

PRM runs were performed using the following gradient of buffer A to buffer B. The composition of buffer A is 5% ACN and 0.1% formic acid in Millipore water. Buffer B is composed of 80% ACN and 0.1% formic acid in water. Peptides were separated by LC-MS using a 100 min gradient composed of buffer A (5% ACN/95% water/0.1% formic acid) and buffer B (80% ACN/20% water/0.1% formic acid) over the following segments: 1–5 min: 1–6% buffer B. 5–75 min: 6–33% buffer B. 75–80 min: 33–100% buffer B. 80–85 min: 100% buffer B. 85–90 min: 100–1% buffer B. 90–100 min: 1% buffer B. The flow rate was 500 nL/min. Technical and biological CVs for each peptide were below 20%, except for VPTANVSVVDLTCR, which exhibited a CV of 22% between runs (Table S1). It has been demonstrated that a higher resolving power for MS2 scans provides only marginal benefit when coming at a cost of slower cycle times or decreased sensitivity.^49^ However, having considered that a resolving power of 7500 for MS2 runs might not be adequate to avoid interference, we confirmed that CVs of select peptides were not meaningfully affected when analyzed at a 60,000 MS2 resolving power, using unscheduled runs to maintain maximal points per peak (Table S1).

### GAPDH Activity Assay

GAPDH enzymatic activity in cell lysates was measured using an assay kit (Sigma-Aldrich MAK277) according to the manufacturer’s protocol. Three 10 cm plates of HEK293T cells were treated with 1 mM propachlor for 30 min in serum-free media, and three 10 cm plates of HEK293T cells were treated with 0.1% DMSO vehicle in serum-free media for 30 min. Each plate was then harvested by scraping with DPBS, and pellets were frozen in −80 ^o^C for future use. Each pellet was lysed in the GAPDH assay buffer, and 8 μL from each plate was aliquoted into a separate row for 4 wells where each well had 42 μL of GAPDH assay buffer including GAPDH Developer. There were 6 rows used in the plate. 2 wells were treated with the GAPDH substrate, and 2 wells were treated without the substrate. Measurements were recorded on a Bio-tek Synergy H1 microplate reader. Absorbance measurements were recorded at 450 nm and normalized to protein concentration measured by Bradford assay.

### PARK7 Deglyoxylase Activity Assay

PARK7 (DJ-1) deglyoxylase assay was modified from Tsumoto et al.^50^ 6 cm plates of HEK293T cells were grown to an 80% confluency, followed by pretreatment with either 1 mM propachlor or vehicle (DMSO) for 30 min in serum-free media. The media was then changed to complete media with or without glyoxal for 2 h, followed by immediate harvest by scraping with DPBS and lysis in 9 parts of RIPA buffer (150 mM NaCl, 50 mM Tris pH 7.5, 1% Triton X-100, 0.5% sodium deoxycholate, 0.1% SDS) and 1 part of 10× PIC for 30 min on ice. Cell lysates were quantified by Bradford assay and loaded on 10% SDS-PAGE gels for Western blotting analysis using anticarboxymethyllysine (CML) antibody. Densitometry over the entire lane was used to quantify the extent of CML modification.

### Protein Aggregation Studies

Three 10 cm plates of HEK293T cells were treated with 1 mM propachlor for 30 min in serum-free media, and three plates were treated with DMSO for 30 min in serum-free media. Media was replaced with complete media for a 6 h recovery. The cells were harvested by scraping in DPBS and lysed in 9 parts of RIPA buffer (150 mM NaCl, 50 mM Tris pH 7.5, 1% Triton X-100, 0.5% sodium deoxycholate, 0.1% SDS) and 1 part of 10× PIC for 30 min on ice. Lysates were separated from cell debris by centrifugation at 21,000*g* for 15 min at 4 °C. The protein in the lysate was quantified by the Bradford assay. Protein samples were diluted to 2 mg protein in 1 mL of RIPA buffer, and an aliquot was removed to represent the total (T) protein prior to ultracentrifugation. The samples were then placed in a TLA-55 rotor and spun in a Beckman Opti-MAX at 77,000*g* for 4 h at 4 °C. The supernatants (soluble fraction (S)) were removed from the pellets. The pellets were washed four times with RIPA buffer. Each wash included a gentle resuspension for 1 min. The remaining dry pellets (P) were resolubilized in 8 M urea in 50 mM Tris pH 7.5 overnight at 4 ^o^C. Aliquots from initial lysis (T), soluble fraction (S), and insoluble fraction (P) were separated by SDS-PAGE, transferred to nitrocellulose, and analyzed by immunoblotting.

Two separate TMT experiments were used to quantify changes in aggregation between propachlor and control-treated samples. TMT sample preparation was adapted from the labeling procedure from AP-TMT-MuDPIT experiments. Only MS quality organic solvents were used during sample preparation. Aliquots of 20 μg were taken from each total (T) sample. Samples were precipitated by methanol/chloroform precipitation. Pellets were then air-dried and resuspended in 1% Rapigest in water. Pellets were visibly completely dissolved. Resuspended protein solutions were then diluted to 50 μL in 100 mM HEPES, pH 8.0, and reduced with 10 mM TCEP for 30 min at 37 °C. Protein solutions were then alkylated with 5 mM iodoacetamide for 30 min in the dark at an ambient temperature. 0.5 μg of sequencing grade trypsin was added to the protein solution for digestion overnight at 37 °C with agitation (600 rpm). TMT isotopic labels were resuspended (100 μg/80 μL ACN), and 40 μL of label was added to each 60 μL sample of digested peptides. Samples were labeled for 1 h at an ambient temperature. Labeling was quenched with 0.4% of ammonium bicarbonate at an ambient temperature for 1 h. Samples were pooled, acidified, and centrifuged for 30 min at 21,100*g* to remove any insoluble debris. Samples were then dried by centrifugal evaporation to 10 μL. Solutions were then brought to 200 μL in buffer A, incubated at 37 °C for 1 h, and centrifuged for 30 min at 21,100*g*. Solutions were transferred to new low-binding tubes (Eppendorf), and the process of heat-spinning was repeated three more times to complete the elimination of Rapigest. Resuspended pellet (P) fractions were quantified by the Bradford assay. A volume was determined that would, on average, yield 20 μg of protein from each of the six samples. That volume of resuspended pellet was then taken from each sample, and proteins were cleaned up and desalted by methanol/chloroform precipitation. The sample was then prepared and labeled as described above for the T fractions.

### Resazurin Assay

For cell viability experiments exploring propachlor treatments, 50,000 cells were plated in 64 wells in a poly-d-lysine-coated 96-well plate. Each well was treated with the indicated concentration of propachlor in serum-free media for 30 min. The media was then changed, and the cells were allowed to recover for 24 h. Two mg of resazurin sodium salt was resuspended in 1 mL of DPBS. 5 μL of this resazurin solution was added to each well, such that the final resazurin concentration was 380 μM. Fluorescence measurements were recorded on a Bio-tek Synergy H1 microplate reader at a 550 nm excitation and 590 nm emission. The bandwidth filter selected for excitation was from 540 to 560 nm. The bandwidth filter for emission was from 580 to 600 nm.

### Activity-Based Protein Profiling (ABPP)

ABPP experiments performed were adapted from others.^51−53^ Three 10 cm plates of HEK293T cells were treated with 1 mM of propachlor for 30 min in serum-free media. Three other 10 cm plates were treated with DMSO for 30 min in serum-free media. Cells were immediately scraped in DPBS after treatment was complete. Cells were lysed in the same RIPA buffer as used in AP-TMT-MudPIT experiments. Lysates were then centrifuged 21,000*g* for 15 min at 4 °C to remove cell debris. Bradford assay was then used to measure 500 μg of protein aliquots. Each protein aliquot was then diluted to 1 μg/nL in RIPA supplemented with protease inhibitors.

The following click chemistry steps described were all performed consecutively. We first added IAA-alkyne to a final concentration of 100 μM and then incubated in the dark for 1 h. We then added azo biotin-azide to a final concentration of 50 μM and vortexed it. We then added TCEP until the solution was 1 mM and vortexed again. We then added TBTA to a final concentration of 100 μM and CuSO_4_ to 1 mM. Samples were rotated at an ambient temperature for 1 h, followed by CHCl_3_/MeOH precipitation. Protein pellets were washed four times with 500 μL of MeOH before drying at an ambient temperature (17–21 °C). Protein pellets were resuspended in 53 μL of 9 M urea in 1% w/v SDS in 1× DPBS by pipetting, diluted to 1 mL with 1× DPBS, bath-sonicated and extruded with a 30-gauge needle, and loaded onto RIPA-washed agarose avidin beads (100 μL beads if settled). Avidin purification was performed overnight at 4 °C. Beads were washed twice with RIPA, twice with 1 M KCl in 0.1% of Triton X-100, twice with 2 M urea in 0.1% Triton X-100 in 50 mM Tris pH 8, and twice with RIPA, followed by drying on a spin column (Pierce). 55 μL of 50 mM sodium dithionite in 1% SDS in 1 × DPBS was added to settled beads. After 1 h, the eluate was collected in low protein-binding tubes (Eppendorf). The elution process was repeated. Two eluates were combined and mixed. 9 μL of eluate was denatured in reducing Laemmli buffer (by adding 3 μL of 4 × Laemmli buffer with 10 mM DTT and boiling for 10 min).

400 μL of MeOH was added to the remnant eluates, mixed well by vortex mixing, followed by the addition of 100 μL of CHCl_3_ and vortex mixing. 300 μL of H_2_O was added to precipitate the protein. After two 5 min of 12,500*g* centrifugation, the upper layer was aspirated without disturbing protein pellets at the liquid interface. Protein pellets were washed four times with 0.5 mL of MeOH, before being air-dried in hood. To the dried protein pellets, 3 μL of 1% w/v RapiGest, 47 μL of 0.1 M HEPES pH 8, 2.63 μL of 0.1 M TCEP were added sequentially and mixed intermittently by flicking the tubes. After incubation for 3 h at 37 °C with 600 rpm agitation, 5.85 μL of 0.1 M iodoacetamide was added to each tube, mixed by flicking and incubated in the dark at an ambient temperature for 1 h. 1 μg of trypsin was added to each tube. Tryptic digestion was performed overnight in the dark at 37 °C with 600 rpm agitation.

Digests were TMT-labeled (50 μg per eluate sample, 1 h, >40% v/v ACN), as indicated in Figure S11, and quenched with 4.14 μL of 10% w/v ammonium bicarbonate at an ambient temperature for 1 h. TMT-6-plex-labeled digests were pooled, and the solvent was removed via vacuum centrifugation (SpeedVac). Pellets were solubilized with 200 μL buffer A. 30 μL of formic acid was added to each sample, followed by heating samples in a 37 °C bead bath for 1 h to hydrolyze RapiGest. Samples were clarified by hardspin for 30 min at 4 °C. Supernatants were transferred to new low protein-binding tubes, heated, and clarified for another two times. P10 Zip-tips with 0.6 μL C18 resin were activated with 500 μL of ACN, washed with 500 μL of 50% ACN (v/v), and twice with 500 μL of buffer A by pipetting slowly back and forth for 10 times each wash with a p20 pipette. 30 μL of each sample was loaded by pipetting slowly back and forth 60 times. C18 resins were washed twice with 500 μL of buffer A by pipetting 5 times each and eluted twice with 50 μL of 50% ACN by pipetting back and forth 15 times. Two eluates were pooled and vacuum-centrifuged. Residues were resuspended in 10 μL buffer A and transferred to 150 μL of polypropylene LC vial insert (Agilent) for injection. Set 5 was analyzed on an LTQ Orbitrap Velos platform with multidimensional protein identification technology (MuDPIT). MS^1^ was performed ranging from 110 to 2000 *m*/*z*, with a resolving power of 30,000 in the orbitrap, followed by MS/MS acquisition starting at 100 *m*/*z*, with an isolation window 1 Da, fragmented by HCD with a normalized collision energy of 45%, detected in orbitrap with a resolving power of 7500. Sets 1–4 were analyzed on a Fusion Lumos Tribrid platform with a high-field asymmetric waveform ion mobility spectrometer (FAIMS) attached prior to the ion transfer tube. The internal stepping compensational voltage was set as −40, −60, and −80 V. MS^1^ scan was performed ranging from 400 to 1500 *m*/*z*, with a resolving power of 60,000 in the orbitrap, a 50 ms maximum injection time and automated gain control target at 400,000, followed by MS/MS acquisition starting at 120 *m*/*z*, with an isolation window of 0.5 Da by quadrupole, fragmented by HCD with a normalized collision energy of 38%, detected in orbitrap with a resolving power of 50,000, a 200 ms MS^2^ maximum injection time, and an AGC target at 50,000.

## Results and Discussion

Cellular stressors induce cellular remodeling through changes in transcription and translation,^30,54^ and consequent changes in protein levels would complicate Hsp40 affinity measurements. To isolate acute protein destabilization effects, we exposed HEK293T cells to chloroacetanilides for only 30 min.^55^ Acute herbicide treatments at high (but sublethal) concentrations are often used to mimic the effects of chronic exposure at low doses.^15,56−58^ In line with previous acute exposure studies, we exposed HEK293T cells to 1 mM of each chloroacetanilide in serum-free media for 30 min.^15^ This concentration and exposure time of acetochlor slightly induce the HSR target HSPA1A in DNAJB8^H31Q^-expressing HEK239T cells, indicating protein misfolding, but no evidence of HSR activation was observed for the other herbicides (Figures S1 and S2, Supplemental Discussion).

The Hsp40 affinity approach is described in Figure 1B. ^Flag^DNAJB8^H31Q^ was transiently overexpressed in HEK293T cells, followed by 30 min of chloroacetanilide herbicide treatment (or vehicle) and immediate Flag immunoprecipitation from the cellular lysate. Coimmunoprecipitated proteins were tryptically digested to peptides, labeled by isobaric tandem mass tags (TMTs)^59^ and then identified and quantified by LC/LC-MS/MS. Each herbicide treatment (acetochlor, alachlor, and propachlor) was performed over 12 biological replicates (with 12 matching vehicle controls) and analyzed by four 6-plex TMT runs (Figure S3). The overall protein distribution in eluates, as analyzed by SDS-PAGE separation followed by silver staining, does not show gross differences between the immunoprecipitates, as expected from such a short treatment (Figure S4).

**Figure 2:**
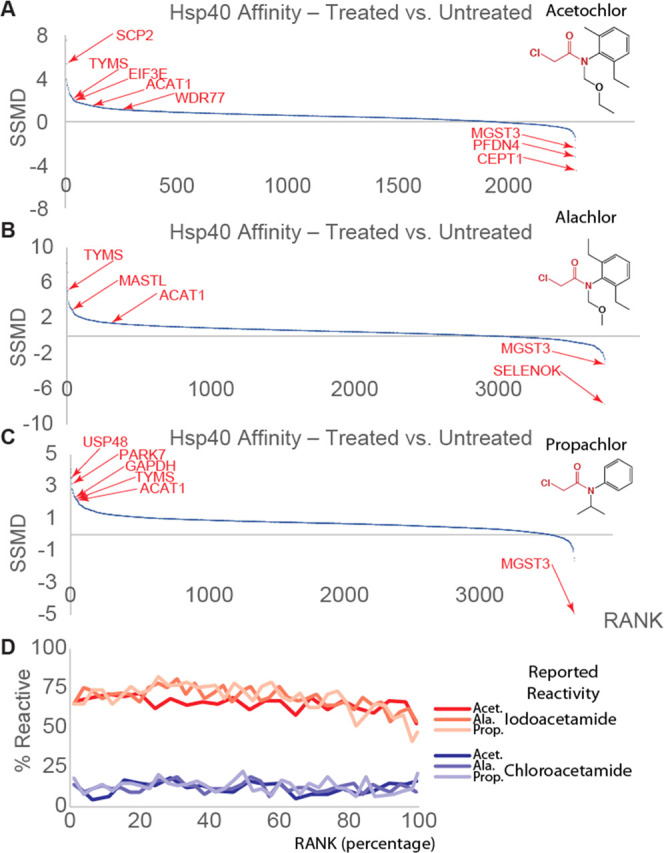
(A–C) Differential Hsp40 affinity of proteins in response to treatment (1 mM, 30 min in serum-free media, *n* = 12 biological replicates) of HEK293T cells with the indicated herbicides. The DNAJB8^H31Q^-interacting proteins are ranked along the *x*-axis by strictly standardized mean differences (SSMDs, variance-normalized differences between control and treatment, similar to a *z*-score). Notable proteins are indicated by red arrows. (D) Percent of proteins (binned in groups of 100 according to ranked SSMD) that were reported as reactive (>4-fold loss of recovery with an iodoacetamide probe) to a general iodoacetamide probe or any of 128 chloroacetamide probes in Kuljanin et al.^17^ Volcano plots can be found in Figures S5, S7, and S8.

The Hsp40 affinity profile for each chloroacetanilide exposure provides a distinct fingerprint (Tables S2–S4). Acetochlor treatment increases DNAJB8 affinity for most (82%) proteins identified across the mass spectrometry runs (Figures 2A and S5), with 2% of clients demonstrating a greater than 2-fold increase in affinity. We further compared this profile to a previously reported liver ABPP study of mice exposed to acetochlor by Counihan and colleagues.^15^ Of the 28 mouse liver proteins that lost iodoacetamide reactivity or alkynylated acetochlor reactivity following mouse acetochlor treatment, 20 were identified in our Hsp40 affinity runs, with 6 demonstrating significantly (*q*-value < 0.05) increased Hsp40 affinity (SCP2, ACAT1, PDIA3, NNT, HSPD1, and DLD). Given the differences in the model system (intraperitoneal injection of mice followed by ex vivo liver excision vs human tissue culture) and in the assays (competitive isoTOP ABPP and Hsp40 affinity), it is encouraging that several of the same targets are found in both studies. Despite this commonality, out of the 338 commonly quantified proteins between this work and that of Counihan et al., there is no correlation between the extent to which proteins are destabilized according to the Hsp40 assay and the extent to which they lose iodoacetamide reactivity following acetochlor exposure (Figure S6; *R*^2^ < 0.01). This lack of correlation indicates that our assay is distinct from measuring adduct conjugation.

Several enzymes with active cysteines prone to electrophilic modification have a significantly greater affinity for DNAJB8^H31Q^ after cellular acetochlor exposure. These include thymidylate synthase (TYMS) (fold change = 3, *q*-value = 0.003), an enzyme essential for the production of thymidine nucleotides,^60^ that has an active-site cysteine that is specifically targeted by an electrophilic chemotherapeutic drug.^61^ Another protein with significantly increased Hsp40 affinity following acetochlor treatment is eukaryotic translation initiation factor 3 subunit E (eIF3e) (fold change = 1.96, *q*-value = 0.0004), which plays a role in tumor growth and the hypoxia response.^62^ Acetyl-CoA acetyltransferase 1 (ACAT1; fold change = 2.91, *q*-value = 0.02) was significantly destabilized, consistent with its multiple reactive cysteines.^17,63,64^ It is possible that adduct formation could stabilize rather than destabilize a protein, leading to a decrease in Hsp40 affinity. Choline/ethanolaminephosphotransferase 1 (CEPT1) and microsomal glutathione S-transferase 3 (MGST3) bind less to DNAJB8^H31Q^ after treatment, indicating stabilization. These two enzymes contain active-site cysteines that interact with substrates (ethanolamine phosphate and glutathione, respectively).^65,66^ Alternatively, these proteins could have lower abundance in response to propachlor treatment, though both CEPT1 and MGST3 are fairly long-lived proteins^67,68^ and so would have to be actively degraded to be depleted on the time scale of the experiment. MGST3 is particularly notable in the context of the known detoxification of chloroacetanilides by enzymatic glutathione conjugation.^69−72^ The stabilization of MGST3 could reflect the product inhibition by hydrophobic glutathione conjugates observed for some^73,74^ but not all^75^ of this family of enzymes.

Despite its lack of HSR induction, alachlor exposure increases the DNAJB8 affinity of many more proteins than acetochlor exposure (Figures 2B and S7). 767 proteins show significantly (*q*-value < 0.05) increased DNAJB8 affinity following alachlor exposure, as opposed to 81 proteins following acetochlor exposure. Selectively targeted proteins included microtubule-associated serine/threonine kinase like (MASTL) (fold change = 2.02, *q*-value = 5 × 10^–5^), a kinase involved in mitosis,^76^ and zinc finger protein 24 (ZNF24) (fold change = 1.89, *q*-value = 0.04), a tumor suppressor.^77^ The greater impact of alachlor as opposed to acetochlor is in some ways surprising, as the two molecules are isomers differing only by the location of a methyl group. However, regioisomers can have substantially different lipophilicities and cellular uptake.^78^ Alachlor has a log *K*_ow_ of 3.5, as opposed to 3.0 for acetochlor. Small structural differences have been shown to have large effects on protein reactivity for other electrophilic series.^79^ The stronger protein destabilization response to alachlor treatment could also reflect the differential metabolism of the compounds. Alachlor is less reactive to glutathione in plants than acetochlor.^71^ Compared to alachlor, acetochlor metabolizes faster into 2-chloro-*N*-(2,6-diethylphenyl)acetamide (CMEPA) and 2-methyl-6-ethylaniline (MEA).^80,81^ While the hepatic microsomal pathway is not available in the HEK293T cells, other chloroacetanilide decomposition pathways could be available.

Propachlor treatment has the strongest effect on the DNAJB8^H31Q^-associated proteome (Figures 2C and S8 and Table S4), with 1765 proteins having increased DNAJB8 affinity. The two most prominent targets that are unique to propachlor are glyceraldehyde-3-phosphate dehydrogenase (GAPDH; fold change = 5.97, *q*-value = 6.09 × 10^–5^) and Parkinson’s disease protein 7 (PARK7/DJ-1, fold change = 3.8, *q*-value = 5.56 × 10^–6^). GAPDH is an enzyme that canonically uses a pK-perturbed active-site cysteine to bind and reduce nicotinamide adenine dinucleotide (NAD) in glycolysis^82^ but consistent with its high abundance also engages in extensive moonlighting activities.^83^ Due to its high abundance and pK-perturbed active-site cysteines, GAPDH is a frequent conjugation target of electrophilic molecules.^84,85^ GAPDH is found in Parkinson’s disease (PD)-associated aggregates, and its aggregation is promoted by electrophilic conjugation.^86^ Given its abundance and fold change in the DNAJB8 co-IP recovery, it is likely that GAPDH is the feature observed by silver stain at 37 kDa (Figure S4). Thermodynamically destabilized PARK7 variants are linked to familial Parkinson’s disease,^87^ and PARK7 overexpression protects against the chemical induction of Parkinson’s phenotypes.^88^ Although a wide variety of biochemical mechanisms have been ascribed to PARK7, including chaperoning and proteolytic activities,^89^ the evidence is strong that it serves as an oxidative stress sensor that protects against cysteine oxidation,^90^ as well as a cellular deglycase preventing electrophilic protein damage.^91−95^

The most straightforward mechanism by which electrophile exposure destabilized proteins is through direct conjugation to reactive protein sites. Consistent with this mechanism, most affected proteins are iodoacetamide-reactive, suggesting that direct conjugation at reactive cysteines is the primary mechanism of protein destabilization due to cellular chloroacetanilide exposure (Figures 2D and S9). If inherent protein reactivity is the main determinant of protein destabilization, we would expect that proteins with the highest inherent electrophilic reactivity would also be those most destabilized. To evaluate this hypothesis, we compared the ranked SSMDs with respect to Hsp40 affinity for all identified proteins against reactivity profiles reported in Kuljanin et al.^17^ We found no correlation between the promiscuity of proteins for chloroacetamide modification and their destabilization from the Hsp40 affinity assay for any of the three chloroacetanilide exposures that we profiled (Figure 2D). This suggests that the reactivity between individual chloroacetanilides and individual proteins is based upon the chemical properties of the molecules and not the inherent reactivity of the nucleophile or electrophile. Furthermore, for each chloroacetanilide herbicide, about one-third of destabilized proteins are not iodoacetamide-reactive. These proteins could be subject to other mechanisms of destabilization, such as conjugation with secondary metabolites^96−98^ or loss of chaperoning due to a global loss of proteostasis capacity.^6,99,100^ We did search for cysteine-chloroacetanilide adducts from each run, finding 32/8637, 40/16029, and 102/15694 conjugated/total peptides following acetochlor, alachlor, and propachlor treatments respectively. Modified peptide identification in the absence of enrichment is typically low, as stoichiometry on a per-peptide basis for post-translational modifications is often low.^101^ Furthermore, when peptide identifications are filtered, the filtration thresholds are set for each search such that the average false discovery rate based on a decoy set is <1% of peptides. While that intended threshold is likely reasonably close to the true false discovery rate in the context of the entire proteome,^15^ for a modification found on only a small number of modified peptides it introduces a much higher practical risk of misidentification.^102^ With these caveats in mind, we include the list of identified chloroacetanilide conjugates in Table S5. It is also worth noting that we consistently see a DNAJB8 modification at C70. While this is in the DNAJB8 J-domain,^103,104^ which is dispensable for client binding, we cannot rule out the possibility that this modification itself could impact the DNAJB8 recognition.

**Figure 3:**
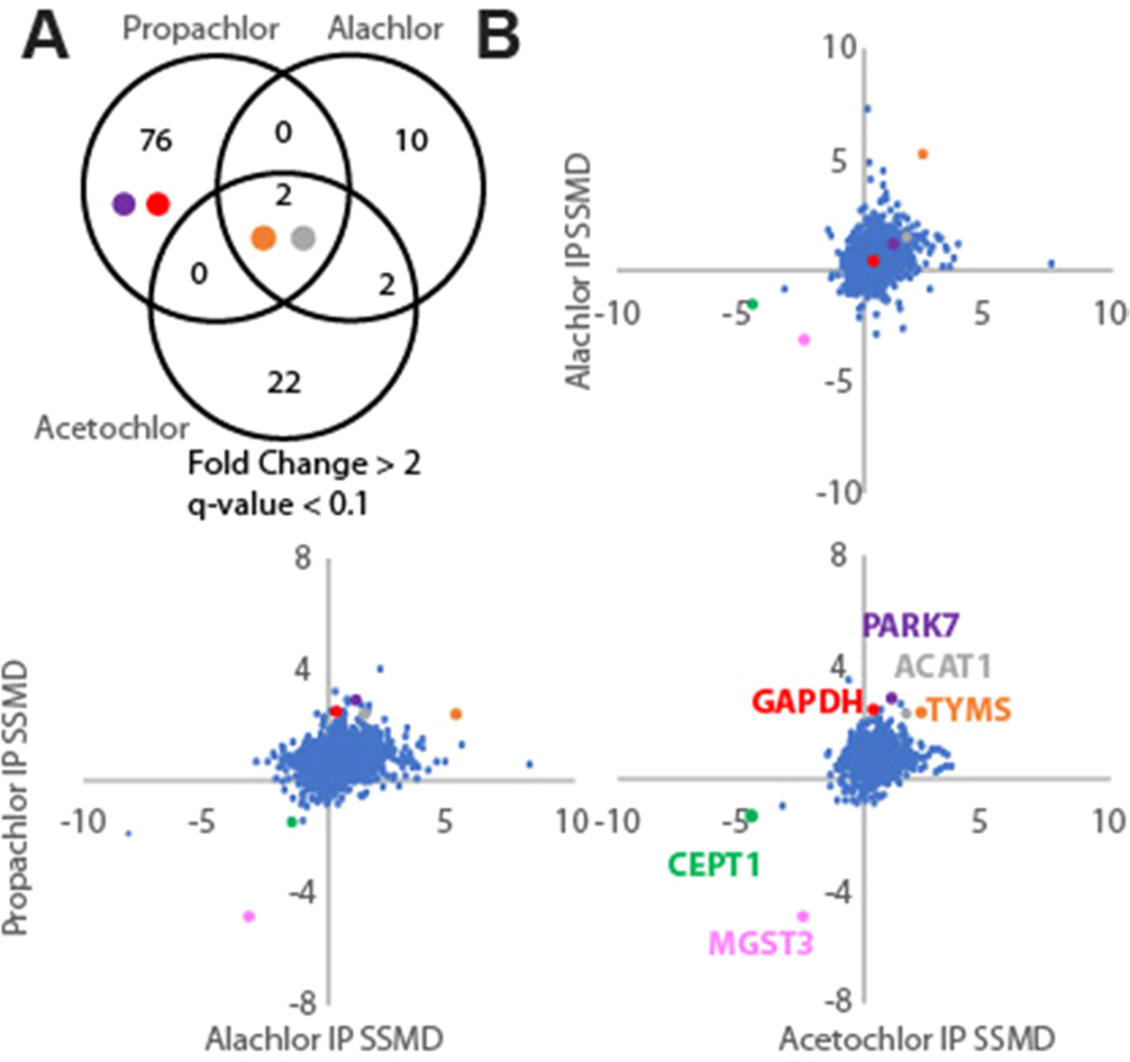
Comparison of proteome-wide Hsp40 affinity changes from the three chloroacetanilide herbicide treatments. (A) Comparison of the most impacted proteins and (B) comparison of strictly standardized mean differences (SSMDs; treatment vs control). Coloring of points in this figure is used consistently to show relative reactivities of PARK7, ACAT1, TYMS, GAPDH, CEPT1, and MGST3.

Including PARK7 and GAPDH, 78 proteins have substantially increased (fold change > 2 and *q*-value < 0.1) affinity for DNAJB8 following propachlor treatment (Figure 3A). This is greater than twice the combined number of proteins destabilized under the same criteria after alachlor and acetochlor treatments. The higher susceptibility of the proteome to propachlor could be based on substitution reactivity. Kinetic studies between propachlor and alachlor reactivities found a 2-fold increase in the substitution of propachlor against several nucleophiles and a lower Gibbs free energy required for substitution reactions of propachlor with nucleophilic thiols.^105^ All three treatments cause a marked destabilization of TYMS and ACAT1 and an apparent stabilization (decreased DNAJB8 affinity) of CEPT1 and MGST3 (Figure 3B). Outside of those, the proteins that are most affected by each individual treatment are unique. This selectivity is particularly salient given the high concentrations (1 mM) used for the treatments. The unique profiles of proteins affected by each herbicide are consistent with the high selectivity of protein reactivity with even strongly reactive chemical warheads.^106−108^ The protein target spectrum of these herbicides cannot be determined by reactivity alone and rather must be evaluated for each individual exposure agent.

**Figure 4:**
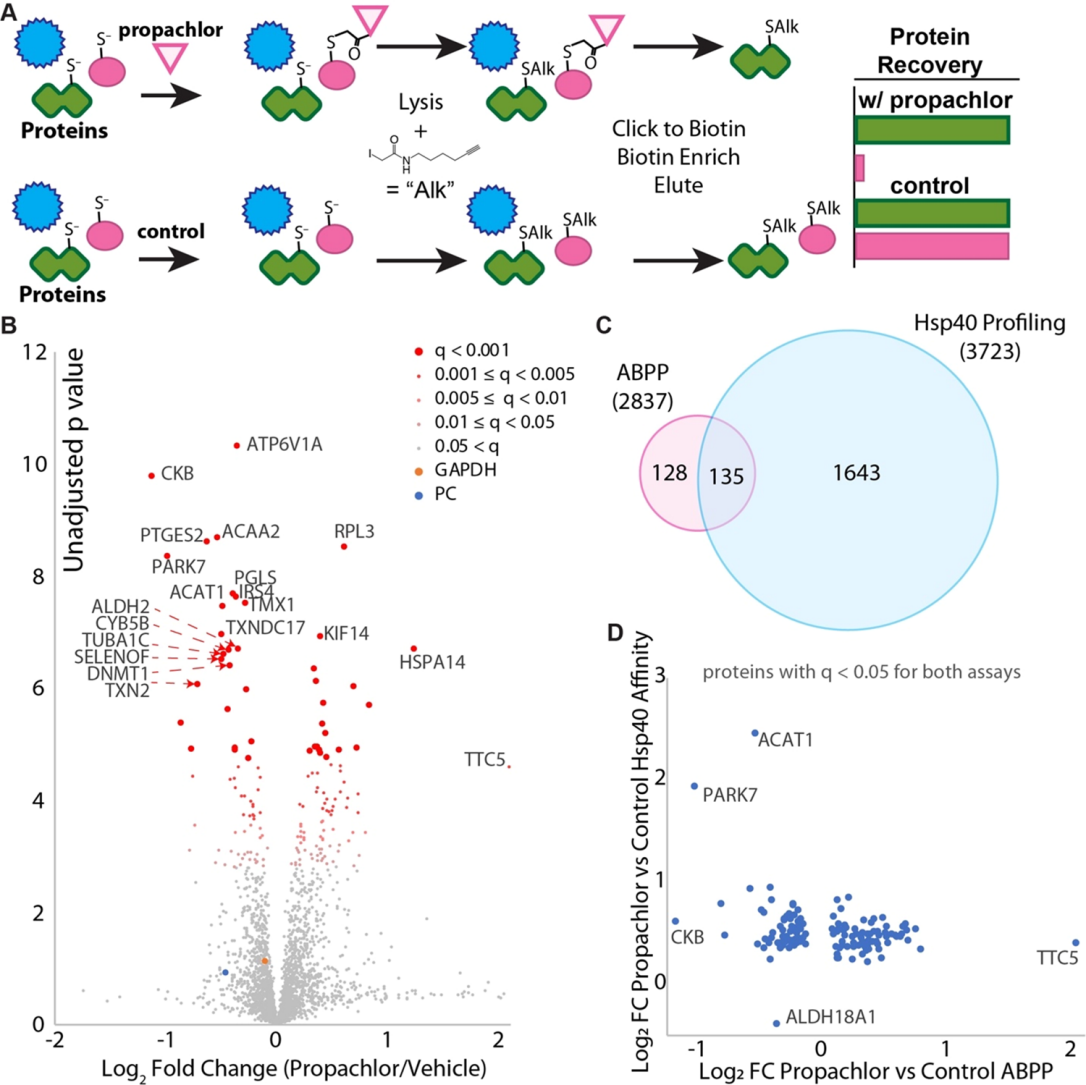
Comparison of Hsp40 affinity and competitive activity-based protein profiling (ABPP) for identification of propachlor targets. (A) Schematic illustrating the ABPP method. (B) Volcano plot illustrating proteins with different iodoacetamide conjugations due to propachlor treatment (*n* = 15 biological replicates with matched vehicle controls). *q*-values were determined using Storey’s modification of the Benjamini–Hochberg approach. (C) Venn diagram comparing significant (*q* < 0.05) protein targets determined by either ABPP or the Hsp40 affinity approach (confer Figure S9 for the Hsp40 affinity profiling volcano plot). The number in parentheses indicates the total number of proteins quantified across at least one TMT-6-plex run. (D) Comparison of fold changes for proteins (*n* = 135) that were identified as significant targets by both assays. There is only a weak negative correlation (slope = −0.2, *R*^2^ = 0.08).

To better determine whether Hsp40 affinity profiling is complementary or just redundant to ABPP for chloroacetanilides, we performed competitive ABPP using the same cellular propachlor treatment conditions as those for which we performed Hsp40 affinity profiling (Figure 4A). 15 plates of cells each were treated with propachlor (1 mM, 30 min) or vehicle in serum-free media and immediately lysed. Lysates were treated with alkynyl-iodoacetamide, conjugated to azide-azo-biotin^109^ in the presence of copper, avidin-purified, reductively eluted with dithionite, and analyzed by quantitative proteomics using TMT (Figures S10 and S11). 2837 proteins were quantified in at least 6 samples, with a global false discovery rate (Storey π_0_) of 0.68. 263 proteins were identified as having significantly changed recovery from cells that were propachlor-treated (*q* < 0.05), with 113 showing decreased recovery following propachlor treatment and 150 demonstrating increased recovery (Figure 4B). About half (135) of these significantly affected proteins were also identified as having significantly different stability from the Hsp40 affinity assay (Figure 4C), while only about 8% (135/1778) of significantly affected proteins from the Hsp40 affinity assay are identified from ABPP. For example, both assays find PARK7, ACAA2, and ACAT1 as highly significant targets (Figure 4D), while only the Hsp40 affinity assay identified GAPDH and TYMS as targets. Even for proteins identified by both assays, there is no clear correlation between the strength of the hit between the assays (Figure 4D). For example, creatine kinase CKB is the strongest hit from ABPP (Figure 4D) but is a relatively weak hit from Hsp40 affinity profiling. GAPDH is a strong hit from Hsp40 profiling but propachlor has no effect on GAPDH recovery using the ABPP probe (Figure 4D).

**Figure 5:**
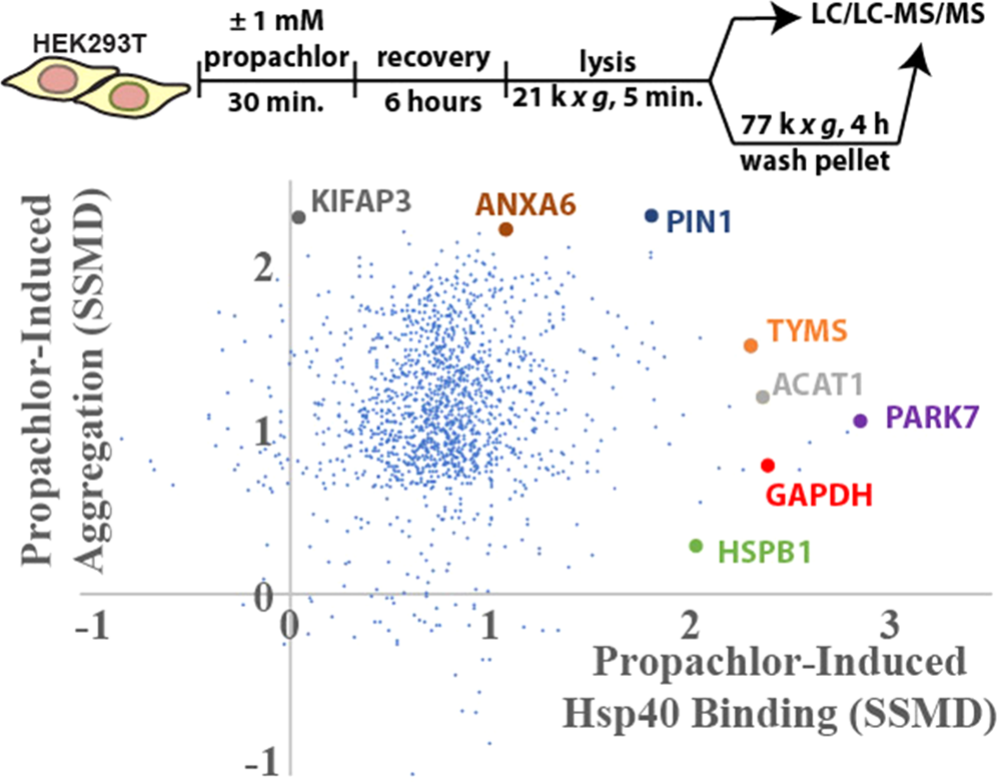
Aggregation of cellular proteins in response to propachlor exposure. Strictly standardized mean deviations (SSMDs) for propachlor-dependent protein insolubility and Hsp40 affinity. HEK293T cells were treated as indicated, lysed, preclarified by centrifugation, and the lysates were normalized to total protein. Protein aggregates were further prepared by ultracentrifugation (6 biological replicates for each treatment condition). The plot compares the change in aggregate levels for each protein to the change in Hsp40 binding (from Figure 2C) for proteins identified in both sets of experiments (1477 proteins). Volcano plots for both propachlor-dependent changes in the total and aggregated proteome are in Figures S12 and S13.

Protein misfolding is a necessary intermediate step for protein aggregation,^110,111^ and all misfolded proteins have the potential to aggregate.^112^ We investigated whether propachlor exposure prompts misfolded cellular proteins to aggregate. We used ultracentrifugation to isolate the insoluble proteome following cellular propachlor exposure (Figure 5 and Table S6). Cells were allowed to recover 6 h post-treatment to allow misfolded proteins time to partition toward an aggregated state. Propachlor treatment has no significant effect on lysate protein abundances, indicating that the cellular proteome is not meaningfully remodeled over this time scale (Figure S12). Despite no change in protein abundance, nearly all detected proteins aggregate more in response to cellular propachlor exposure (Figures 5 and S13). However, as with Hsp40 affinity profiling and the ABPP assay, there is also no correlation between the change in Hsp40 affinity and the increase in aggregation. These proteins may not be well-surveyed by a promiscuous Hsp40 such as DNAJB8, even under conditions that lead to their destabilization. Alternatively, they may have lower thresholds for aggregation as compared to proteins that show greater differential DNAJB8 affinity following propachlor treatment.^113^ This lack of correlation is consistent with previous findings that protein solubility does not correlate with structural changes.^23^

GAPDH is highly abundant in the human proteome with many functions beyond its canonical role in glycolysis.^82,83^ These functions are readily perturbed by a diverse range of post-translational modifications,^114^ which can lead to toxic aggregation,^115^ making its destabilization following propachlor exposure particularly hazardous for cellular proteostasis. GAPDH is susceptible to modification by a wide range of electrophiles, including 4-hydroxynonenal and methylglyoxal,^116,117^ which in turn regulates its function.^118,119^ Cysteines 152 and 156 in the GAPDH NAD+ binding site are particularly subject to electrophilic modification.^120−122^ Despite it being a hit only from the Hsp40 affinity profiling and not by ABPP, we looked for the presence of GAPDH-propachlor adducts.

**Figure 6:**
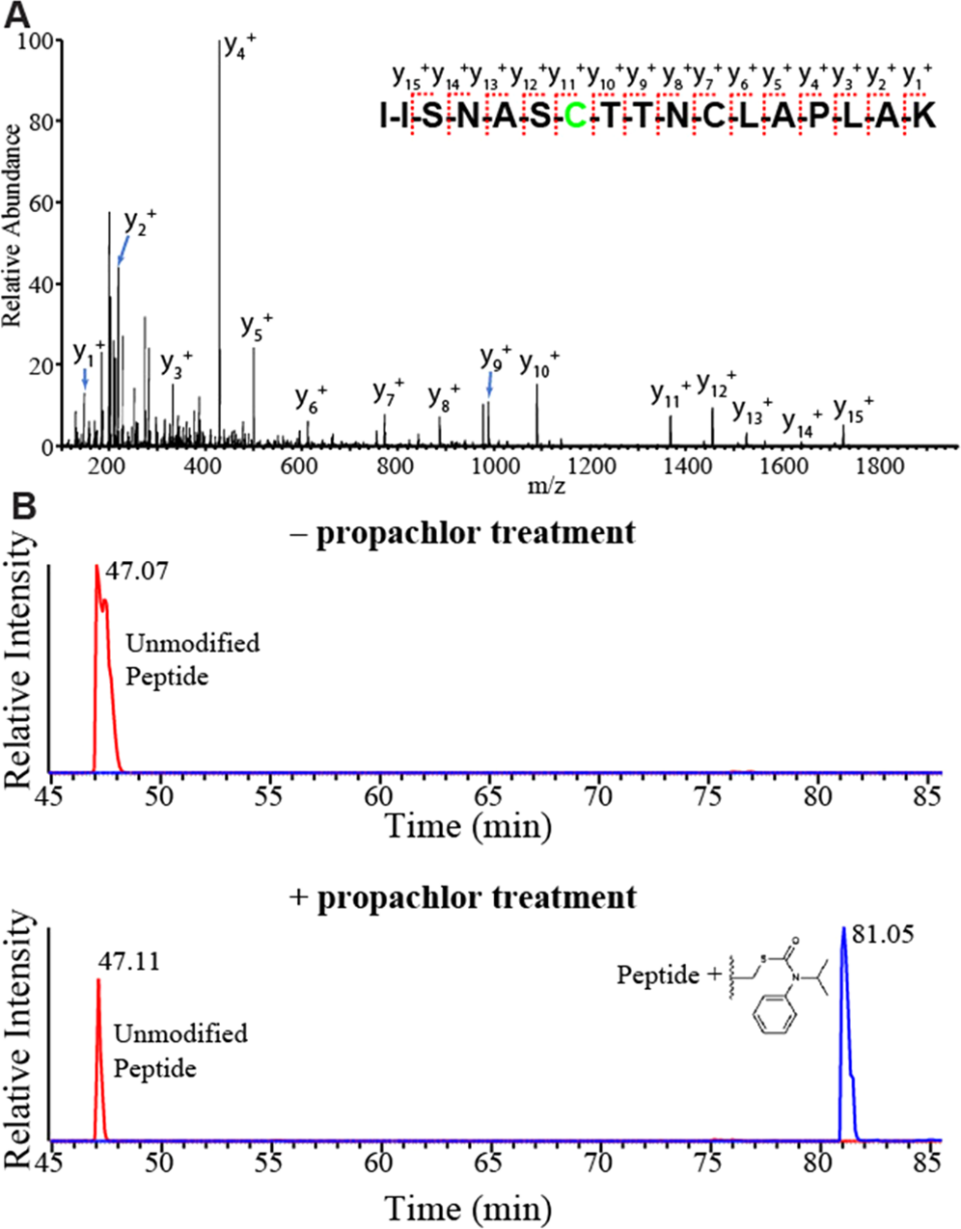
Propachlor modifies a catalytic cysteine in the active site. (A) MS2 fragmentation spectrum obtained from the LC-MS/MS shotgun proteomics analysis of lysate collected from propachlor-treated (1 mM, 30 min, serum-free media) HEK293T cells. This peptide is modified at the C152 position with an adduct that corresponds to propachlor thiocarbamate. C156 is carbamidoylated by iodoacetamide. (B) PRM chromatograms demonstrating the dependence of the adduct on propachlor treatment.

To determine whether GAPDH is modified during propachlor treatment, we immunoprecipitated ^Flag^GAPDH from lysates following cellular propachlor treatment. GAPDH interactions are interrupted by our stringent RIPA washes, so that ^Flag^GAPDH was prepared with minimal contamination by other proteins (Figure S14A). In the absence of treatment, GAPDH was primarily present as the N-terminally acetylated protein, with a smaller population of the glutathione conjugate (Figure S14B). After propachlor treatment, a new base peak appeared at M+176 Da, consistent with a single propachlor adduct (Figure S14C). No evidence of multiple propachlor adducts was observed. We further analyzed immunoprecipitated ^Flag^GAPDH by digestion and shotgun proteomics, followed by an open adduct search.^44^ The propachlor modification is clearly localized to C152 based on MS2 spectra (46 total spectral counts) (Figure 6). C152 is in the NAD^+^ binding site and is necessary for catalytic activity.^123^ Across two biological duplicates, we find a 26 ± 7% drop in the unmodified C152 intensity, implying that about a quarter of GAPDH is modified. No evidence for modification at C156 was observed. The specificity of propachlor modification for the C152 site, while leaving C156 as the free thiol, explains why Hsp40 affinity identifies GAPDH as a target of propachlor while competitive ABPP is unable to do so (Figure 4D). Propachlor treatment does not affect the availability of the highly reactive C156 for iodoacetamide modification, leaving GAPDH readily recovered by ABPP in both conditions.

To understand how global stability of GAPDH is impacted by propachlor treatment, we profiled the rest of the protein using targeted limited proteolysis (LiP).^30^ Destabilized protein domains are more extended and thus more susceptible to cleavage by a promiscuous protease, such as thermolysin or proteinase K (PK).^124^ LiP involves the brief treatment of lysate to protease followed by shotgun proteomics to characterize the yield of cleavage events.^20,125,126^ Loss of a tryptic peptide indicates a protein conformational change in the vicinity of that peptide sequence.^127^

**Figure 7:**
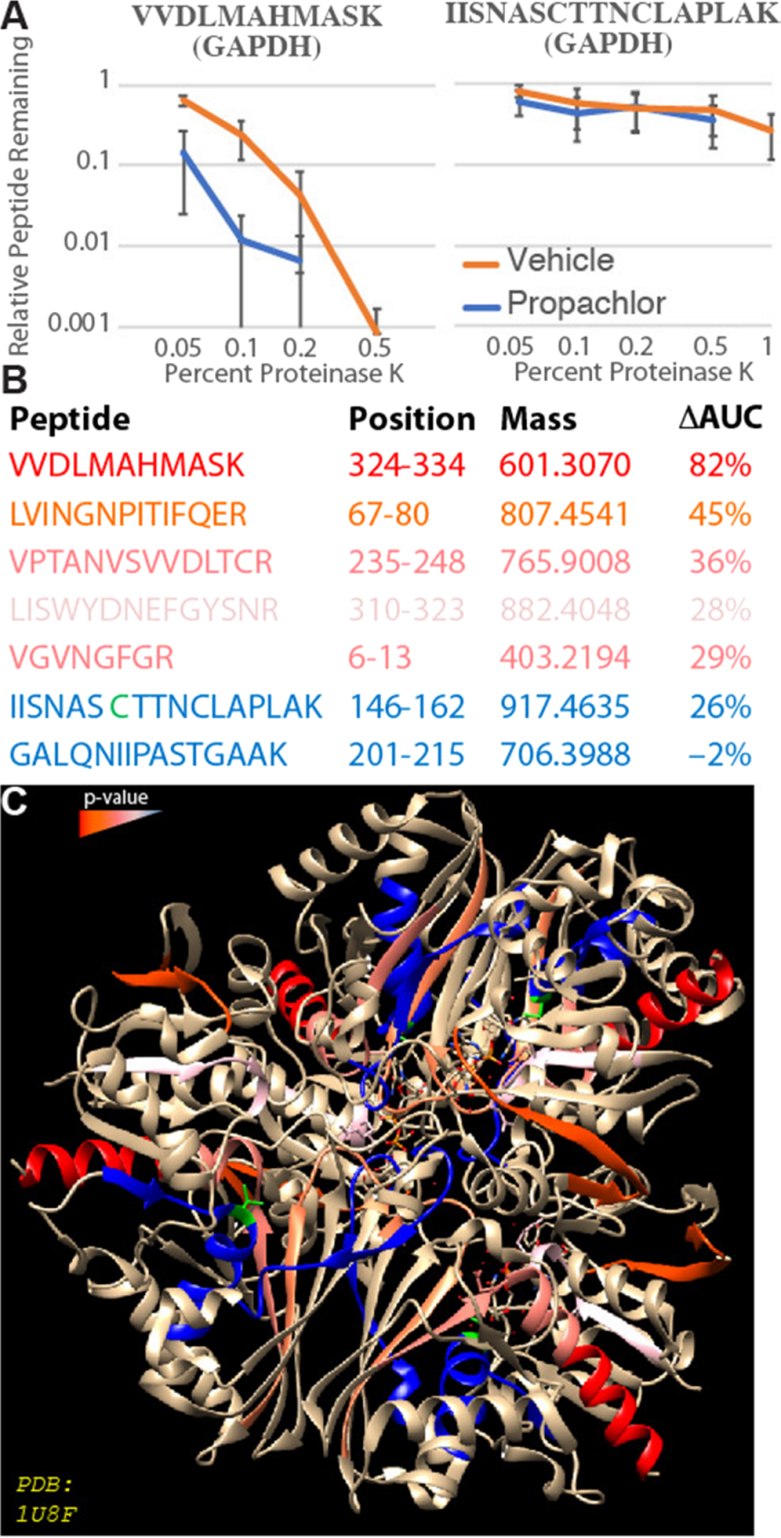
Propachlor destabilization of GAPDH peptides measured by LiP. (A) LiP-PRM traces illustrating the proteolytic susceptibility of two GAPDH peptides following cellular treatment with propachlor (blue) or vehicle (orange) as indicated. (B) Characteristics of the analyzed GAPDH peptides. ΔAUC refers to the decrease in the area under the curve for the proteolytic susceptibility curves. (C) The GAPDH peptides are colored according to the significance of the effect of propachlor treatment on proteolytic susceptibility. C152 is indicated in green. The structure (PDB: 1U8F) is taken from Jenkins et al.^129,130^ PRM chromatograms are in Figure S20.

We selected peptides from GAPDH for LiP to assess structural changes after propachlor treatment. We found several GAPDH peptides to be more proteolytically sensitive to proteinase K after propachlor treatment (Figures 7 and S15), including the active-site peptides LVINGNPITIFQER, LISWYDNEFGYSNR, VGVNGFGR. Hence, propachlor induces a more extended conformation in GAPDH, consistent with destabilization. Destabilization of GAPDH could also affect protein–protein interactions. One of the destabilized peptides, VPTANVSVVDLTCR, is involved in the dimer interface of GAPDH.^128^ Mutations in this peptide are associated with conformational changes at the dimeric interface and a loss of tetrameric stability.^115^ Destabilization of VPTANVSVVDLTCR in GAPDH by propachlor exposure may inhibit the active conformation and affect binding partners. No meaningful change in proteolytic susceptibility was observed for IISNASCTNCLAPLAK, which encompasses the propachlor adduct site. Since this peptide can only be observed in GAPDH that has not been directly modified by propachlor, this implies that the stability of the unmodified GAPDH is not generally perturbed by the treatment. We also attempted LiP on PARK7 peptides. Only three peptides proved suitable for LiP (Table S1), and none showed evidence of differential proteolytic susceptibility following propachlor treatment (Figure S15B). While this indicates that the protein as a whole is not destabilized, it does not exclude the possibility that unprofiled regions of the protein might be affected.^125^

**Figure 8:**
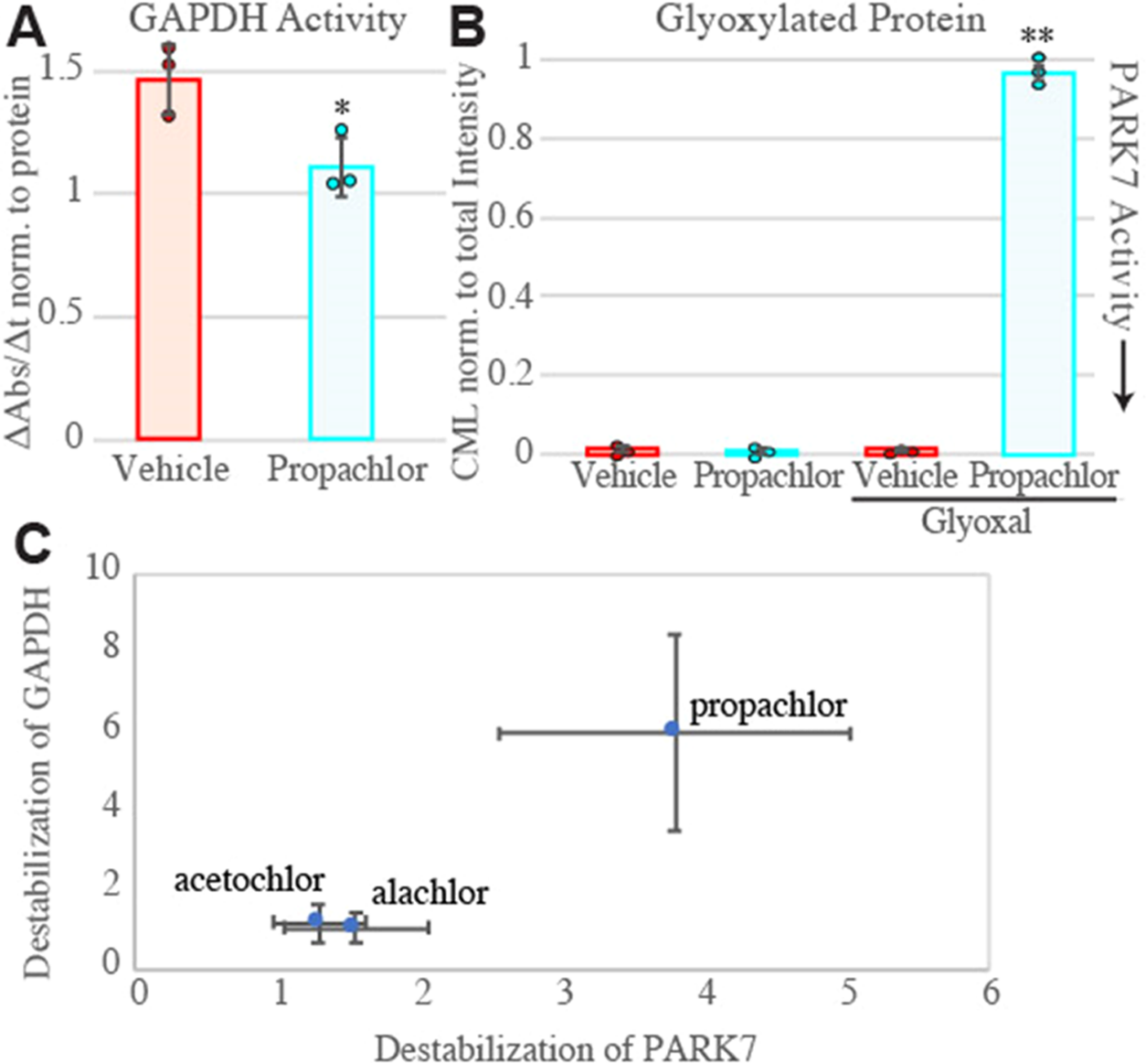
(A) Activity of GAPDH from cells treated with propachlor or vehicle. Activity was determined from the NADH production rate in lysates, as measured by colorimetry at 450 nm over the linear range, and normalized to total protein (g/mL) as determined by Bradford assay. *p* < 0.05 by Student’s two-tailed *t*-test (*n* = 3). Kinetic traces are in Figure S16A. (B) Inactivation of PARK7 determined by total anticarboxymethyllysine (CML) densitometry of SDS-PAGE separated lysates. HEK293T cells were treated for 30 min with vehicle or propachlor (1 mM in serum-free media), followed by 2 h of treatment with vehicle or glyoxal (4 mM) and immediate lysis (*n* = 3). 2-way ANOVA yields F = 1909 > Fcrit = 4.07, and Tukey’s HSD finds propachlor + glyoxal condition mean differences compared to all other conditions exceed the *q*_crit_ for 0.001. (C) Relative Hsp40 affinities of GAPDH and PARK7 following the three cellular treatments. Error represents standard deviation. One data point for GAPDH after alachlor treatment was removed as an outlier using Grubb’s test (*G* = 2.55 > *G*_0.95_ = 2.11, *n* = 9).

Protein destabilization can lead to both gain-of-function (through proteotoxic conformations) and loss-of-function. GAPDH activity has previously been shown to decrease in response to methylglyoxal and copper exposures, presumably due to conjugation and oxidation, respectively.^24,117^ GAPDH modification can further lead to misfolding and aggregation.^131,132^ We evaluated GAPDH activity in cells treated with propachlor. GAPDH activity in lysates was measured using a colorimetric assay for NAD^+^ reduction in the presence of substrate. Treating HEK293T cells with 1 mM propachlor for 30 min decreased the GAPDH activity by 25% (Figure 8A). GAPDH inhibition is associated with the accumulation of its substrate glyceraldehyde-3-phosphate, which can convert to reactive species that alkylate KEAP1 to induce the oxidative stress response.^133^ Indeed, we see that KEAP1 is significantly destabilized by propachlor exposure by the Hsp40 affinity assay (FC = 1.3, *p* = 0.019, *q* = 0.02) (Table S4). This decrease is consistent with the amount of C152 adducts that we detect by mass spectrometry but low considering the strong negative cooperativity between the two catalytic sites on the GAPDH tetramer.^134^ Our proteomic characterization of propachlor-induced aggregation found an increase in GAPDH aggregation induced by propachlor treatment (FC in aggregates = 4.9, *q*-value = 0.008; Figure S13). Similar results were obtained from Western blot analysis assessing the levels of GAPDH in the pellet fraction after ultracentrifugation (Figure S17A); however, there is no significant depletion of total GAPDH (Figure S17B). From this, we can conclude that although GAPDH destabilization following propachlor treatment does lead to an increase in the aggregated fraction, the total burden of GAPDH aggregation on the cell remains small compared to the high levels of the soluble protein.

PARK7 is a chaperone-like peptidase that can repair proteins damaged by a series of aldehyde products, including methylglyoxal and glyoxal. PARK7 specifically protects proteins, including GAPDH, from cysteine and lysine adducts, including glycerate damage caused by metabolic products constitutively generated by GAPDH.^93,135^ PARK7 is significantly destabilized after propachlor treatment and thus could be inactive, preventing it from protecting cells from these damaged products. A cellular assay designed to quantify the ability of PARK7 to prevent glyoxal modification of proteins in HEK293T cells has been previously established.^50^ We measured the ability of endogenous PARK7 to deglycate glyoxal-modified proteins after incubation with 1 mM propachlor for 30 min (Figures 8B and S16B,C). In the presence of propachlor, the intensity of proteins converted to carboxymethyllysine after glyoxal treatment increased significantly in comparison with the control experiment (DMSO vehicle treatment). Cellular exposure to propachlor inhibits PARK7’s ability to protect proteins from glycation, offering an alternative mechanism by which propachlor exposure can induce protein misfolding beyond direct modification. We speculate that it could be beneficial to the cell that GAPDH is inhibited in concert with PARK7, preventing the accumulation of glycating equivalents when the detoxification mechanism is also inhibited. Due to poor reproducibility for profiling the active C106 in PARK7, we were not able to compare whether PARK7^C106^ and GAPDH^C152^ are similarly modified across the range of chloroacetamides investigated in the reported high-throughput screen.^17^ For the three chloroacetanilides in our present study, however, the relationship holds (Figure 8C).

## Conclusions

The occupational risk of PD associated with herbicide exposure has been well established in many studies and diverse populations. Because farmworkers are subject to combination exposures, it is difficult to separate the contributions of individual exposure agents. Two common pesticides, rotenone and paraquat, are known to induce PD-associated phenotypes in cell culture and model organisms, including oxidative stress, mitochondrial dysfunction, and dopaminergic cell death,^136^ with rotenone also directly oxidizing PARK7/DJ-1.^137^ The effects of both rotenone and paraquat are rescued by PARK7 overexpression, indicating that the PARK7 activity determines sensitivity to these pesticides.^88,138−141^ This further suggests that propachlor exposure could potentiate sensitivity to rotenone, paraquat, and other proparkinsonism toxins.

A significant limitation of this study is the high concentrations (1 mM) of herbicides employed and the *in vitro* conditions (treatment of human cells). Further investigation in relevant organismal models and exposure conditions is necessary to determine whether the specific protein targets found reflect in vivo biology. Although short treatments at high concentration are often used to understand the chemistry of exposure, high concentrations can also bias interactions in favor of the most abundant proteins.^64,142^ In that context, it is particularly striking that even with such high concentrations of herbicides, the protein reactivity profiles are distinct.

In summary, we present profiles of destabilized proteomes in response to cellular exposure to three chloroacetanilide herbicides. While some proteins are destabilized by each treatment, the overall profiles from each herbicide exposure are unique. About 70% of targeted proteins are known to be subject to haloacetamide conjugation at cysteine, consistent with adducts being the primary mechanism of destabilization, but the extent of destabilization does not correlate with haloacetamide reactivity, reflecting the distinction between conjugation and stability. Hsp40 affinity profiling is an effective assay for determining the effect of environmental toxicants on the cellular proteome, both distinct from and complementary to existing technologies.

## Supporting Information Available

Immunoblots addressing HSR response to chloroacetanilide treatment and accompanying discussion, cellular viability curves upon propachlor treatment, TMT labeling assignments, silver stains of Hsp40 affinity profiling eluates, volcano plots for Hps40 affinity profiling, including breakdowns between the iodoacetamide-reactive and noniodoacetamide-reactive proteome, silver stains for ABPP eluates, volcano plots for total and insoluble protein levels following cellular propachlor treatment, intact protein mass spectra for GAPDH following cellular treatments, proteolytic susceptibility curves for GAPDH and PARK7 peptides following propachlor treatment, kinetic traces for GAPDH activity, immunoblots demonstrating carboxymethyllysine levels, and representative MS2 chromatograms for peptides monitored by PRM (PDF)
CVs for PRM peptides (Table S1) (XLSX)
Hsp40 affinity TMT-AP-MS results for acetochlor treatment (Table S2) (XLSX)
Hsp40 affinity TMT-AP-MS results for alachlor treatment (Table S3) (XLSX)
Hsp40 affinity TMT-AP-MS results for propachlor treatment (Table S4) (XLSX)
Identified chloroacetanilide peptide conjugates (Table S5) (XLSX)
Whole-cell and insoluble TMT-MS results following propachlor treatment (Table S6) (XLSX)
