# Supplementary material for: Cellular Exposure to Chloroacetanilide Herbicides Induces Distinct Protein Destabilization Profiles
Source: ACS Chem Biol. 2023 Jul 10;18(7):1661–76. doi: 10.1021/acschembio.3c00338 (PMC10367052; doi:10.1021/acschembio.3c00338)
Supplement: Supplementary file 1 — cb3c00338_si_001.pdf [file cb3c00338_si_001.pdf]

Supporting Information for

Cellular Exposure to Chloroacetanilide Herbicides Induces Distinct Protein Destabilization Profiles

*Guy M. Quanrud, Ziqi Lyu, Sunil V. Balamurugan, Carolina Canizal, Hoi-Ting Wu, and Joseph C. Genereux**

Department of Chemistry, University of California, Riverside, CA 9252, USA

*Joseph C. Genereux, E-mail: josephg@ucr.edu

Table of Contents:

S-3,S-5 through S33	Supplemental Figures
S-4	Supplemental Discussion: HSPA1A Induction
S-34	Supplemental References

Supplemental Table 1 is provided as an external file.

Supplemental Table 2 is provided as an external file.

Supplemental Table 3 is provided as an external file

Supplemental Table 4 is provided as an external file.

Supplemental Table 5 is provided as an external file.

Supplemental Table 6 is provided as an external file.

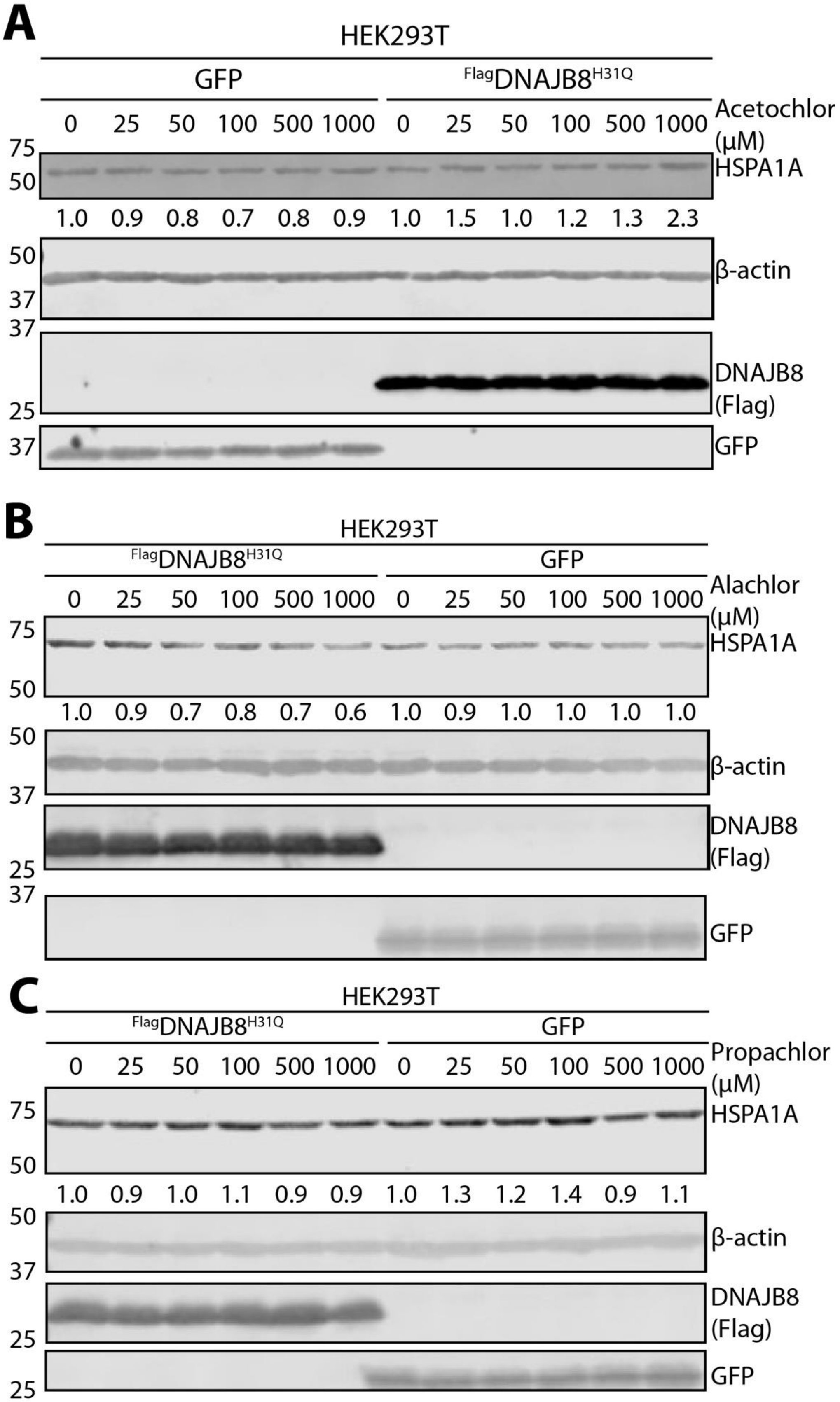
A
HEK293T
GFP
FlagDNAJB8H31Q
0 25 50 100 500 1000 0 25 50 100 500 1000
Acetochlor (μM)
75
50
HSPA1A
1.0 0.9 0.8 0.7 0.8 0.9 1.0 1.5 1.0 1.2 1.3 2.3
50
37
β-actin
37
25
DNAJB8 (Flag)
37
GFP
B
HEK293T
FlagDNAJB8H31Q
GFP
0 25 50 100 500 1000 0 25 50 100 500 1000
Alachlor (μM)
75
50
HSPA1A
1.0 0.9 0.7 0.8 0.7 0.6 1.0 0.9 1.0 1.0 1.0 1.0
50
37
β-actin
DNAJB8 (Flag)
25
37
GFP
C
HEK293T
FlagDNAJB8H31Q
GFP
0 25 50 100 500 1000 0 25 50 100 500 1000
Propachlor (μM)
75
50
HSPA1A
1.0 0.9 1.0 1.1 0.9 0.9 1.0 1.3 1.2 1.4 0.9 1.1
50
37
β-actin
DNAJB8 (Flag)
25
GFP
25

Figure S1: Immunoblots of SDS-PAGE separated lysates from HEK293T cells expressing the indicated proteins and treated with acetochlor (A), alachlor (B), or propachlor (C) at indicated concentrations in serum-free media for 30 min., followed by a 16 h recovery in complete media. HSPA1A density is below blot slices. Molecular weight markers are indicated on the left. Antigens targeted by immunoblotting are listed to the right of the slice.

Supplemental Discussion: HSPA1A Induction

HSPA1A is a transcriptional target of HSF1 activation during misfolded protein accumulation in the cytosol, and hence its accumulation can be used as a proxy for HSF1 activation. To see if there are global changes in protein misfolding load following treatment, we measured HSPA1A levels in the cell as a function of short (30 min.) propachlor exposures in serum-free media. We did not see meaningful changes, except for induction at the highest acetochlor concentration (1 mM) (**Figure S1**) and then only in the presence of DNJAB8^{H31Q}. Hence, in the presence of DNJAB8^{H31Q}, this treatment condition might be adequate to induce a protein misfolding load that is substantial enough to induce HSF1 and HSPA1A upregulation. The absence of HSPA1A induction does not mean that proteins are not misfolding in the other conditions, but rather that it is not adequate to induce the HSF1 transcriptional program[1]. Consistent with this interpretation, propachlor is still toxic to the cells at 1 mM (**Figure S2**). Based on this result, we decided to use 1 mM concentrations throughout the study since we knew that protein misfolding was induced by at least one herbicide at this concentration. Serum-free media is necessary to avoid small molecule scavenging by albumen and is a

standard incubation condition for cellular exposure to acetochlor[2,3,4]. Indeed, the use of complete media ablates HSR activation by acetochlor. The sensitization of cells to HSR by ^{Flag}DNAJB8^{H31Q} expression was not previously observed for HSR induction by arsenite or by cadmium[5], but might relate to increased delivery of misfolded proteins to Hsp70, thus helping titrate Hsp70 off of and activate HSF1[6].

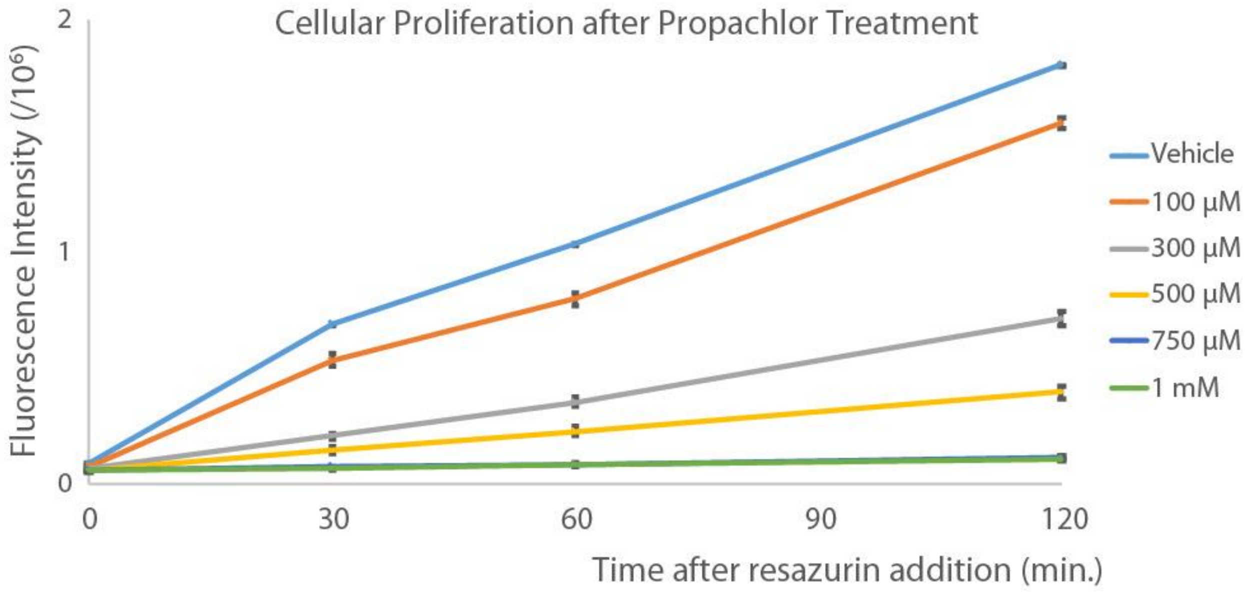

Figure S2: Effect of propachlor on cell growth in HEK293T Cells. HEK293T cells were seeded in 64 wells of a 96 well plate. Each well had 50,000 cells and eight wells were treated with each condition respectively. Standard error is shown as error bars. Each row was treated with the indicated concentration of propachlor in serum-free media for 30 min., followed by a 16 h recovery. Resazurin dye was added to each well and measurements were taken at 0, 0.5, 1, and 2 h post-recovery. Propachlor disrupts cell proliferation of HEK293T cells.

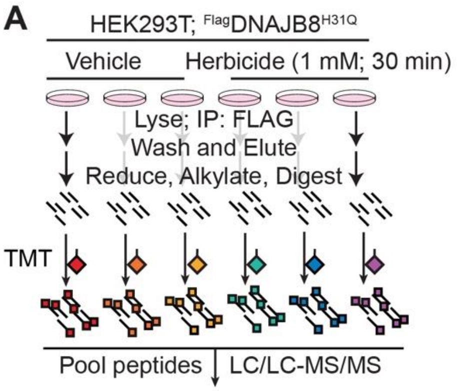

B

MS Run #	Experimental Designation	TMT Channel 126	127	128	129	130	131
1	GQ_VII_44	Veh	Aceto.	Veh	Aceto.	Veh	Aceto.
2	GQ_VII_45	Veh	Aceto.	Veh	Aceto.	Veh	Aceto.
3	GQ_VIII_13	Veh	Aceto.	Veh	Aceto.	Veh	Aceto.
4	GQ_VIII_14	Veh	Aceto.	Veh	Aceto.	Veh	Aceto.
5	GQ_VIII_107	Veh	Ala.	Veh	Ala.	Veh	Ala.
6	GQ_VIII_108	Veh	Ala.	Veh	Ala.	Veh	Ala.
7	GQ_VIII_115	Veh	Ala.	Veh	Ala.	Veh	Ala.
8	GQ_VIII_116	Veh	Ala.	Veh	Ala.	Veh	Ala.
9	GQ_VIII_103	Veh	Prop.	Veh	Prop.	Veh	Prop.
10	GQ_VIII_106	Veh	Prop.	Veh	Prop.	Veh	Prop.
11	GQ_VIII_113	Veh	Prop.	Veh	Prop.	Veh	Prop.
12	GQ_VIII_114	Veh	Prop.	Veh	Prop.	Veh	Prop.

Veh = Vehicle Aceto. = Acetochlor
Ala. = Alachlor Prop. = Propachlor

Figure S3: Experimental Design. A) HEK293T cells are transiently transfected with ^{Flag}DNAJB8^{H31Q}. Cells are treated with vehicle (0.1% DMSO) or herbicide (1 mM for 30 min.) in serum-free media. After treatment, cells are immediately lysed in RIPA buffer with protease inhibitors. Lysates are clarified by centrifugation and pre-cleared over sepharose beads, followed by immunoprecipitation over anti-FLAG beads overnight. Beads are washed well four times with RIPA buffer, and proteins eluted by boiling in Laemmli 6x concentrate. Samples are cleaned up by chloroform/methanol precipitation, reduced, alkylated and by tryptically digested to peptides. Peptides were TMT labeled as indicated in (**B**), followed by pooling and LC/LC-MS/MS analysis.

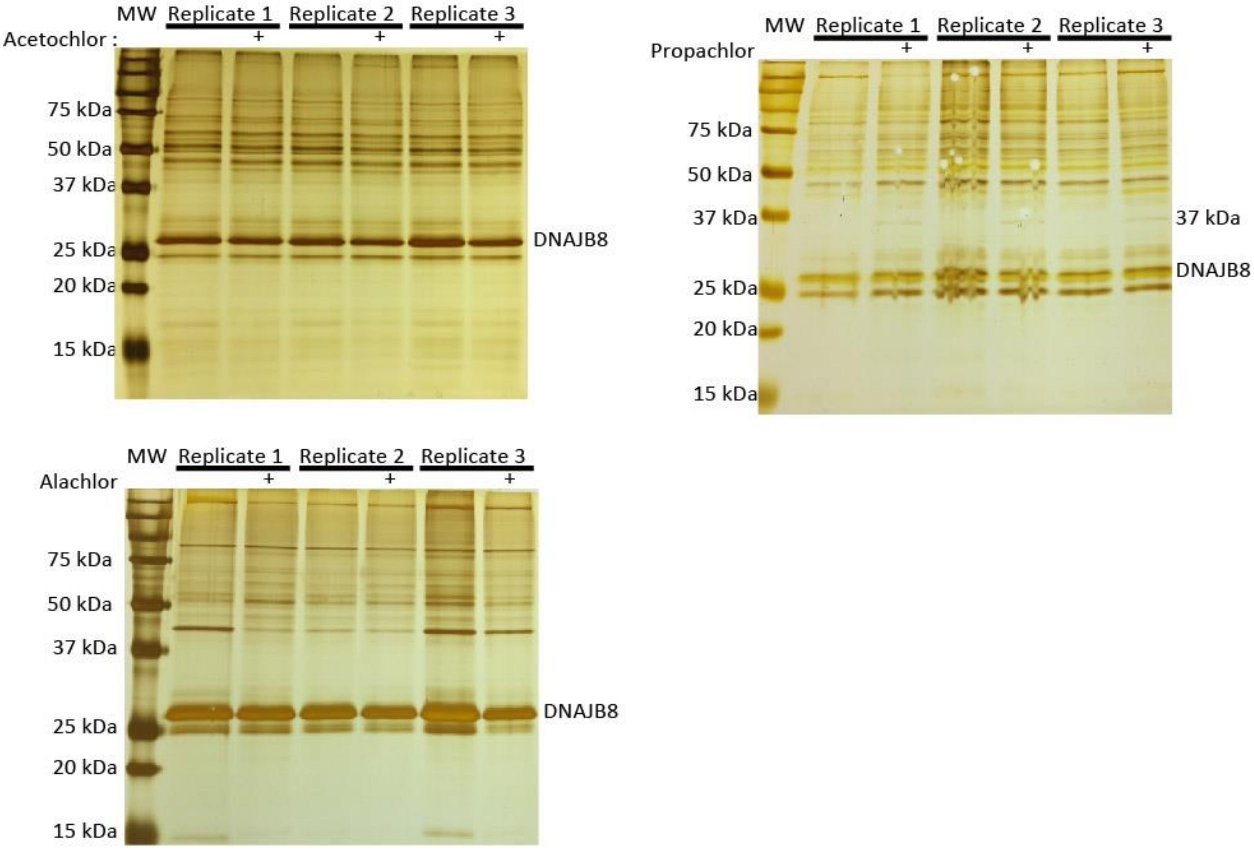

Figure S4: Silver Stains of ^{Flag}DNAJB8^{H31Q} IP eluates. HEK293T cells overexpressing ^{Flag}DNAJB8^{H31Q} were treated with either 1 mM herbicide or 0.1% DMSO (vehicle) in serum-free media. After immunoprecipitation, 15% of eluate were reserved for silver stain. ^{Flag}DNAJB8^{H31Q} is the most abundant protein in each replicate after the immunoprecipitation. Other bands represent proteins recovered with DNAJB8. Only propachlor shows a meaningful difference between treated and untreated conditions: a new band appearing at 37 kDa.

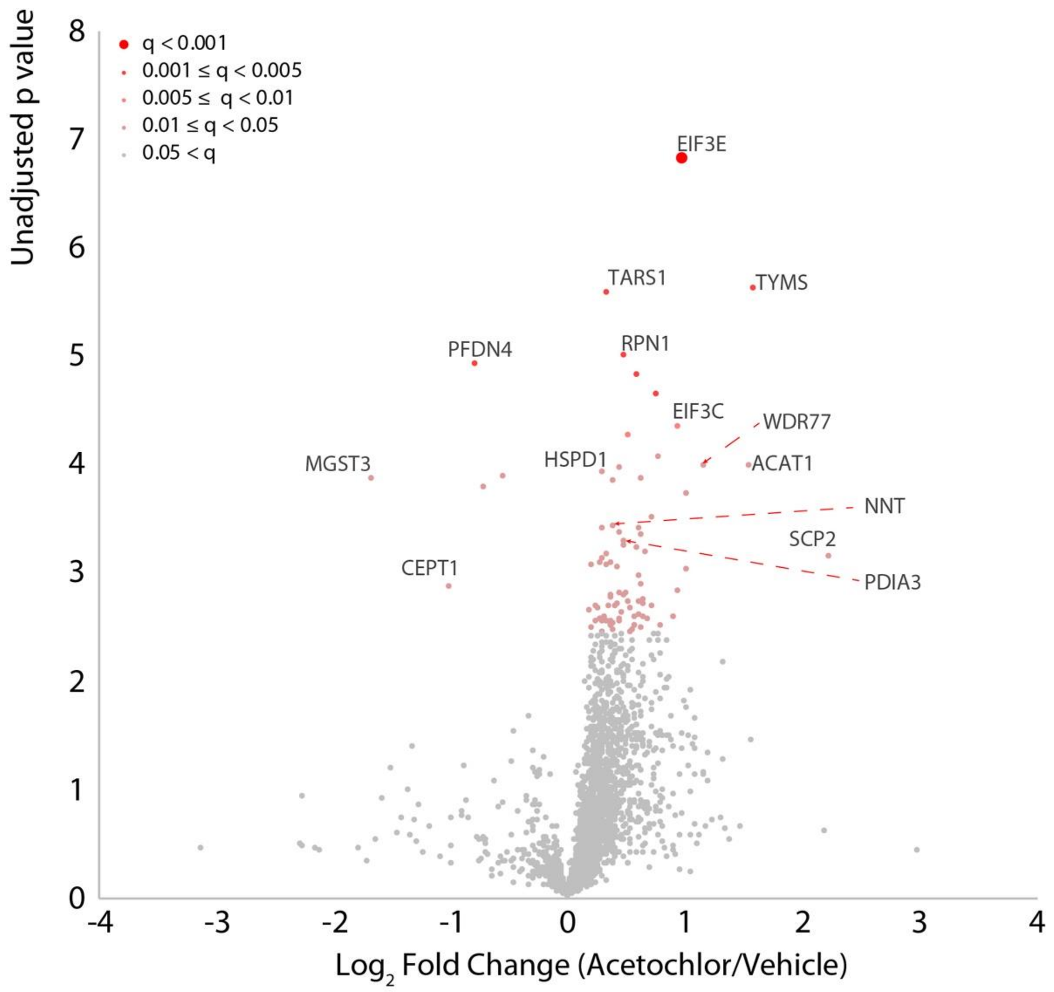

Figure S5: Volcano plot for profiling ^{Flag}DNJAB8^{H31Q} affinity in response to cellular acetochlor exposure. 1 mM acetochlor treatment for 30 min increases the affinity of a subset of proteins with ^{Flag}DNAJB8^{H31Q}. A ^{Flag}DNAJB8^{H31Q} pulldown experiment consists of three transfected HEK293T cells treated with 1 mM acetochlor for 30 min in serum-free media and three plates treated with vehicle (DMSO), followed by immediate lysis. Red dots represent proteins with significantly increased interaction with ^{Flag}DNAJB8^{H31Q}, using a false discovery rate threshold (FDR) of 0.05 (n = 12 biological replicates in 4 TMT-AP-MS runs). Integrated TMT intensities for each protein group and subsequent analysis are provided in **Table S2**.

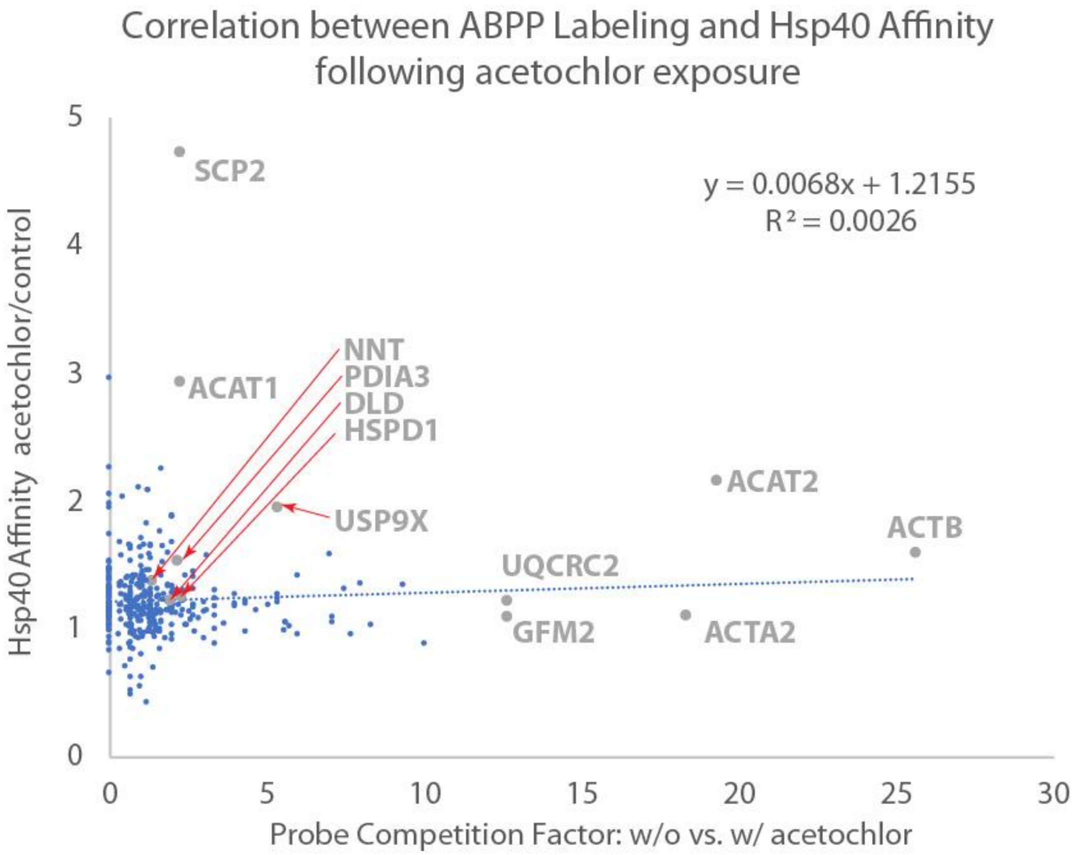

Figure S6: Comparison between ABPP labeling from Table S1 in Counihan et al[1] and Hsp40 affinity (Figure S5) following acetochlor treatment. It must be stressed that Counihan et al treated living mice with acetochlor, followed by iodoacetamide probe labeling of lysed livers, very different from our HEK293T cell incubation.

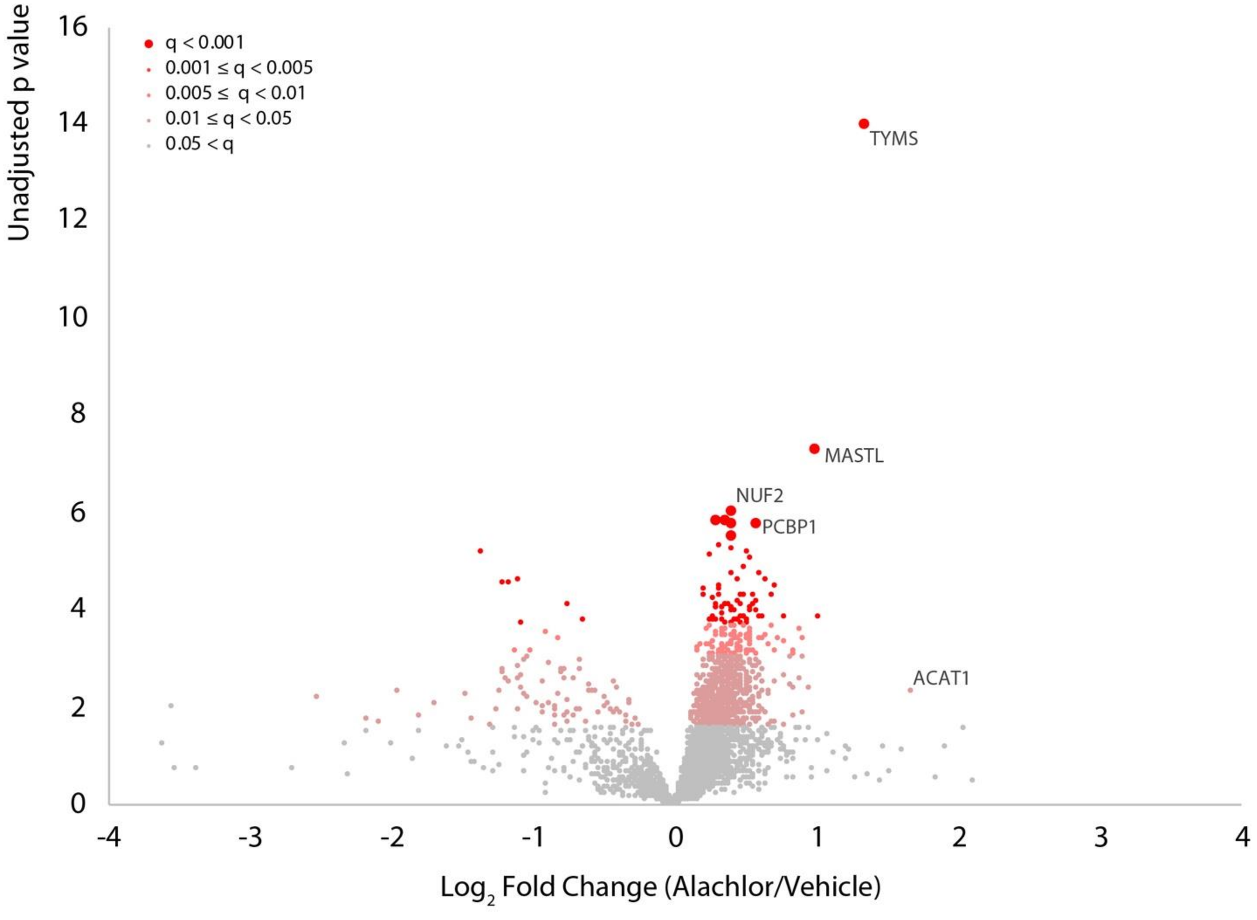

Figure S7. Volcano plot for profiling DNJAB8^{H31Q} affinity in response to cellular alachlor exposure. 1 mM acetochlor treatment for 30 min increases the affinity of a subset of proteins with DNAJB8^{H31Q}. A DNAJB8^{H31Q} pulldown experiment consists of three transfected HEK293T cells treated with 1 mM alachlor for 30 min in serum-free media and three plates treated with vehicle (DMSO), followed by immediate lysis. Red dots represent proteins with significantly increased interaction with DNAJB8^{H31Q}, using a false discovery rate threshold (FDR) of 0.05 (n = 12 biological replicates in 4 TMT-AP-MS runs). Integrated TMT intensities for each protein group and subsequent analysis are provided in **Table S3**. A single data point at $\log_2$FC = 8.8 (FC = 450±540) and –log p = 1.2 is not shown.

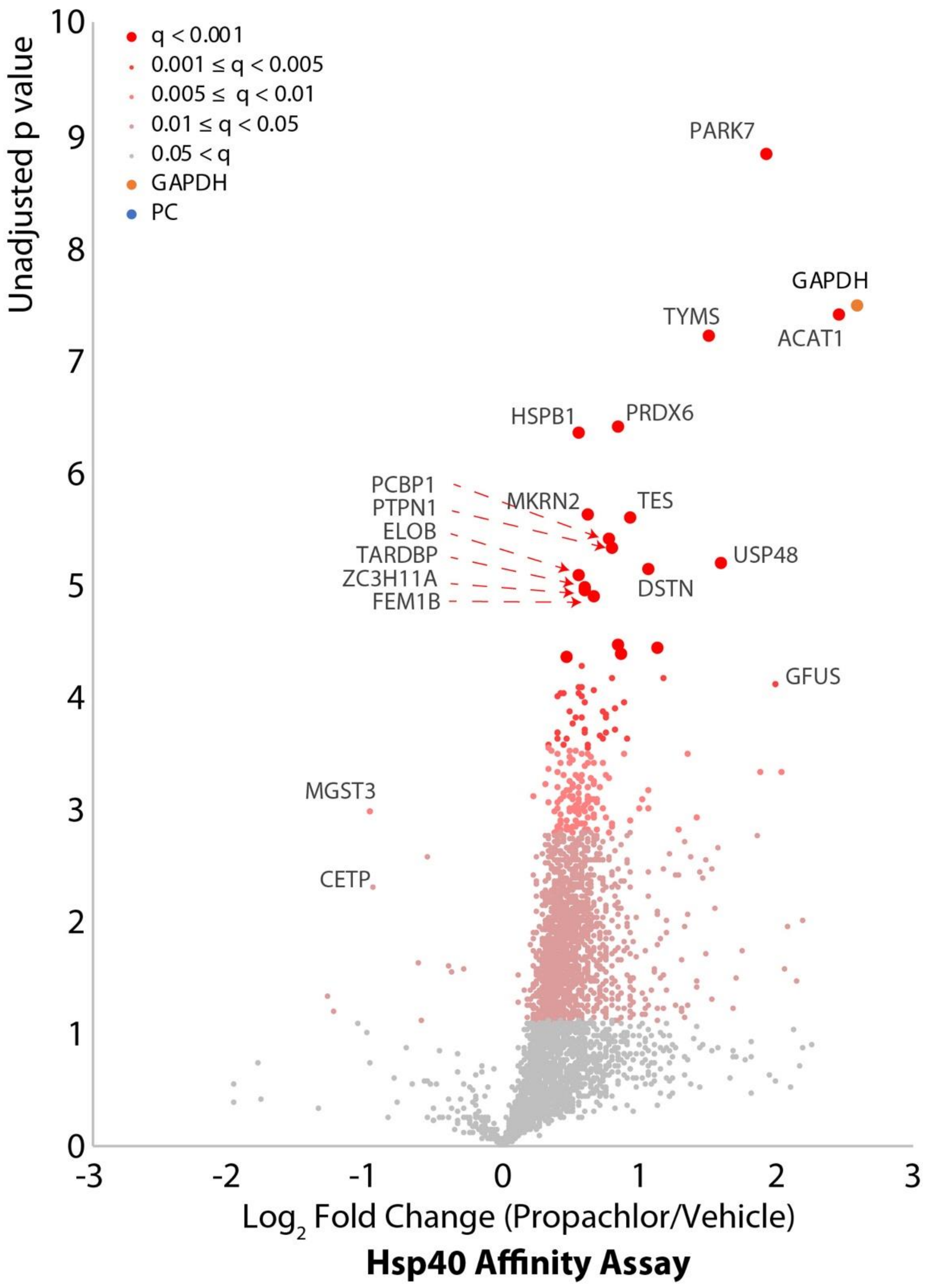

Figure S8. Volcano plot for profiling DNJAB8^{H31Q} affinity in response to cellular propachlor exposure. 1 mM acetochlor treatment for 30 min increases the affinity of a subset of proteins with DNAJB8^{H31Q}. A DNAJB8^{H31Q} pulldown experiment consists of three transfected HEK293T cells treated with 1 mM propachlor for 30 min in serum-free

media and three plates treated with vehicle (DMSO), followed by immediate lysis. Red dots represent proteins with significantly increased interaction with DNAJB8^{H31Q}, using a false discovery rate threshold (FDR) of 0.05 (n = 12 biological replicates in 4 TMT-AP-MS runs). Integrated TMT intensities for each protein group and subsequent analysis are provided in **Table S4**.

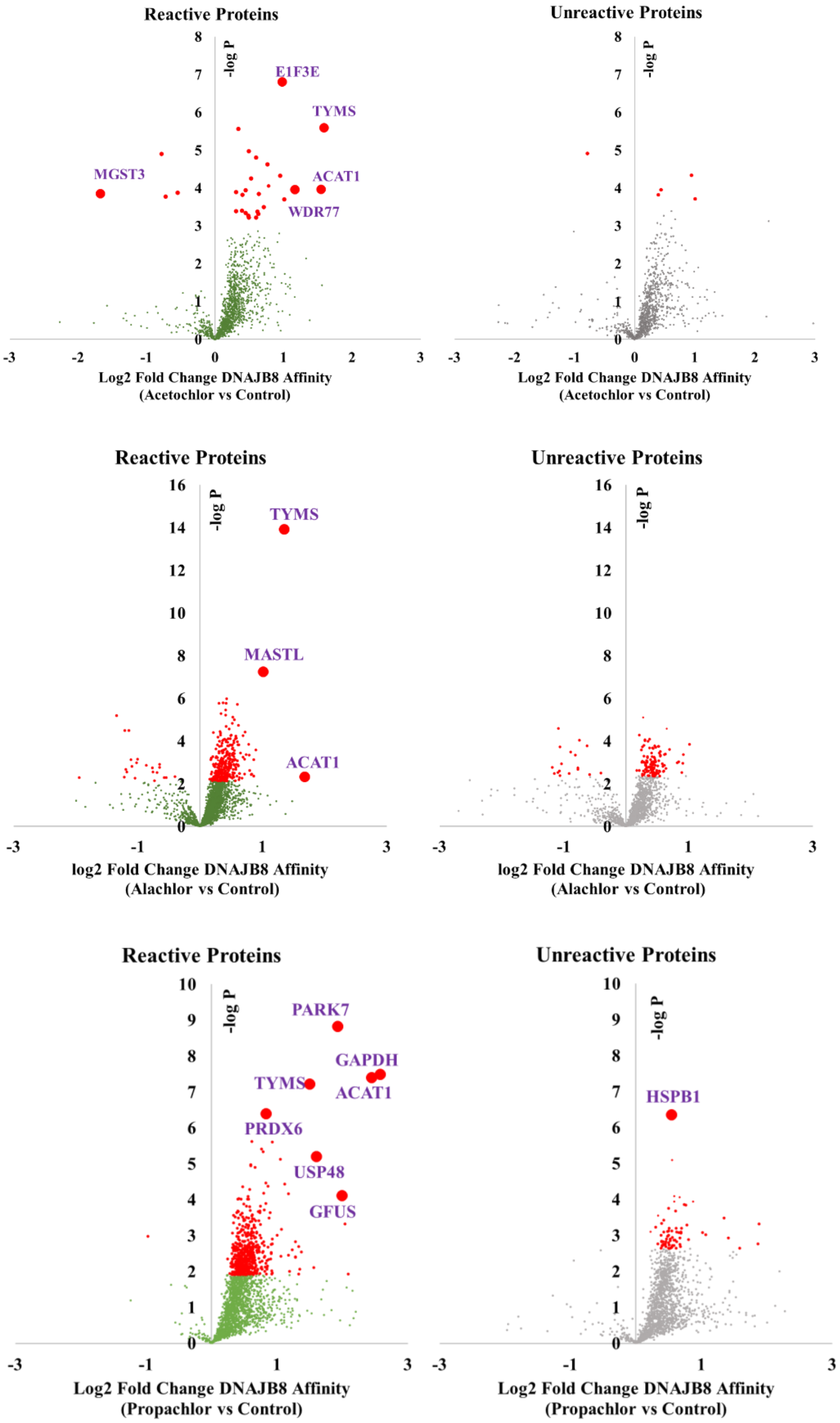

Figure S9: DNAJB8 affinity profiles for iodoacetamide-reactive vs. non-reactive proteins. Iodoacetamide reactivity is based on the electrophilic reactivity library of

Kuljanin et al.[2] Iodoacetamide-reactive proteins are on the left; the remaining proteins are on the right. Red dots represent proteins with significantly increased interaction with DNAJB8^{H31Q}, using a false discovery rate threshold (FDR) of 0.05 (n = 12 biological replicates in 4 TMT-AP-MS runs). Note that although HSPB1 was non-reactive with alkynylated iodoacetamide for the cell lines studied in reference 2, it has been shown in other work to be strongly reactive with iodoacetamide[3].

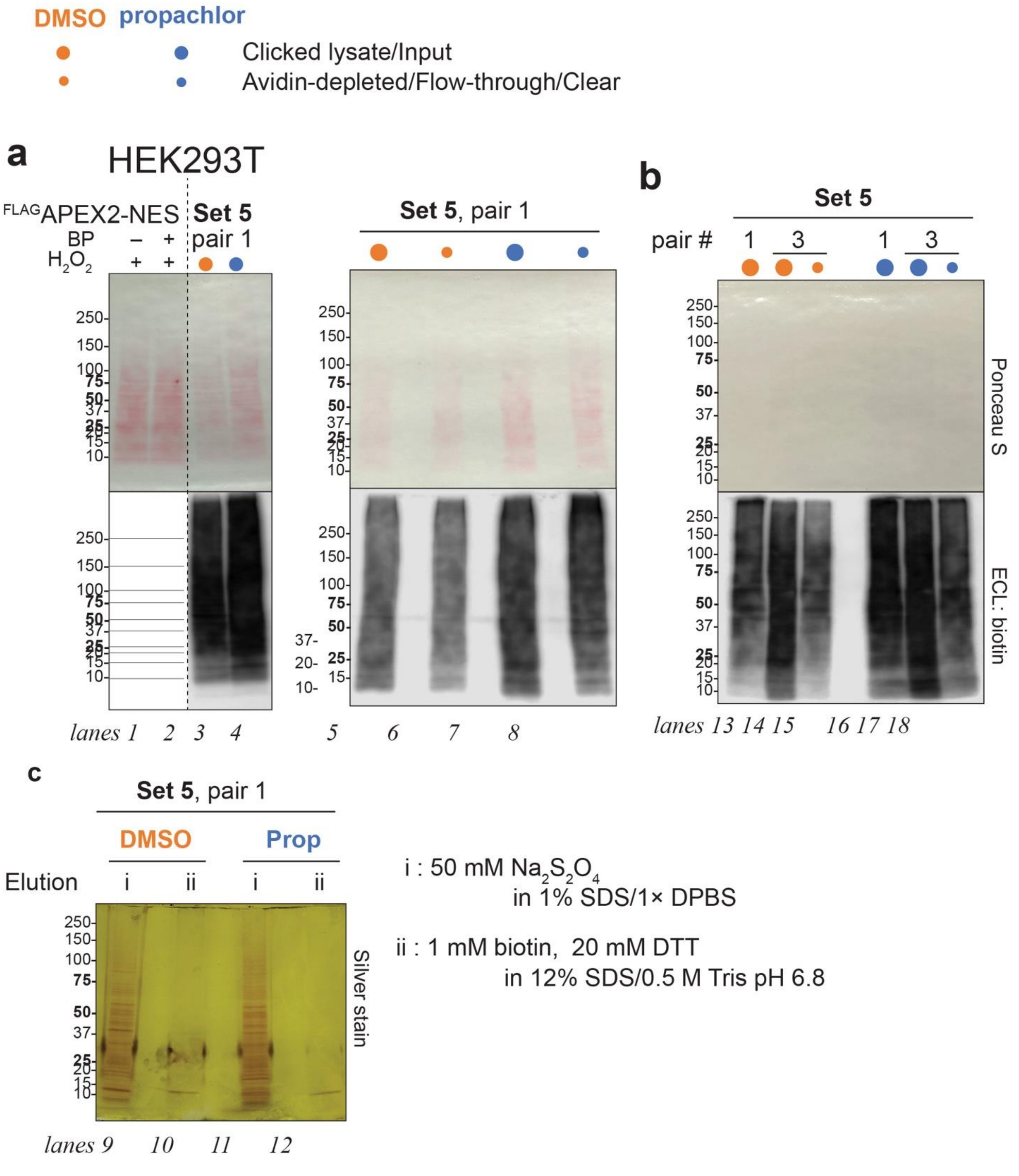

Figure S10: Evaluation of ABPP samples. We used Set 5 to learn iodoacetamide-alkyne labeling efficiency, agarose avidin beads dosability and sodium dithionite elution efficiency. **a**. Based on the 8-fold intensity ratio (lanes 4 vs 2), we initially used 80 µL 50% beads slurry (v/v), or 40 µL settled beads, for 500 µg ABPP lysate protein pellet

resuspension (1 M urea in RIPA). Biotinylated proteins were barely depleted from “clear”s. lanes 6 vs 5, 67% remains; lanes 8 vs 7, 100% remains.

b. Biotinylated protein-bound avidin beads were eluted (I) twice with 55 µL 50 mM dithionite in 1% SDS, and (II) once with 2 mM biotin and 20 mM DTT in 12% SDS. 8.2% of each eluate was analyzed by 4-20% SDS-PAGE, followed by silver staining. 50 mM Dithionite elution alone is enough to recover most biotinylated proteins (lanes 9 vs 10 and lanes 11 vs 12).

c. “Flow-through”s, or “clear”s from Set 5 pair 1 were diluted to a final urea concentration of 0.5 M, and subjected to avidin purification again with 40 µL settled beads. Clicked lysate pellet suspensions from Set 5 pair 3 (0.5 M urea in 0.1% SDS) were loaded onto 40 µL settled beads. 1% “clear” samples were used to check clearance. Remenant biotinylated proteins were still about 40-50% (lanes 15 vs 14 and lanes 18 vs 17, respectively). Another 60 µL settled beads were introduced to each sample for further clearance. Eluates of Set 5 pair 1 samples from a and c were combined accordingly for TMT 6-plex labeling (annotation shown in **Figure S11**).

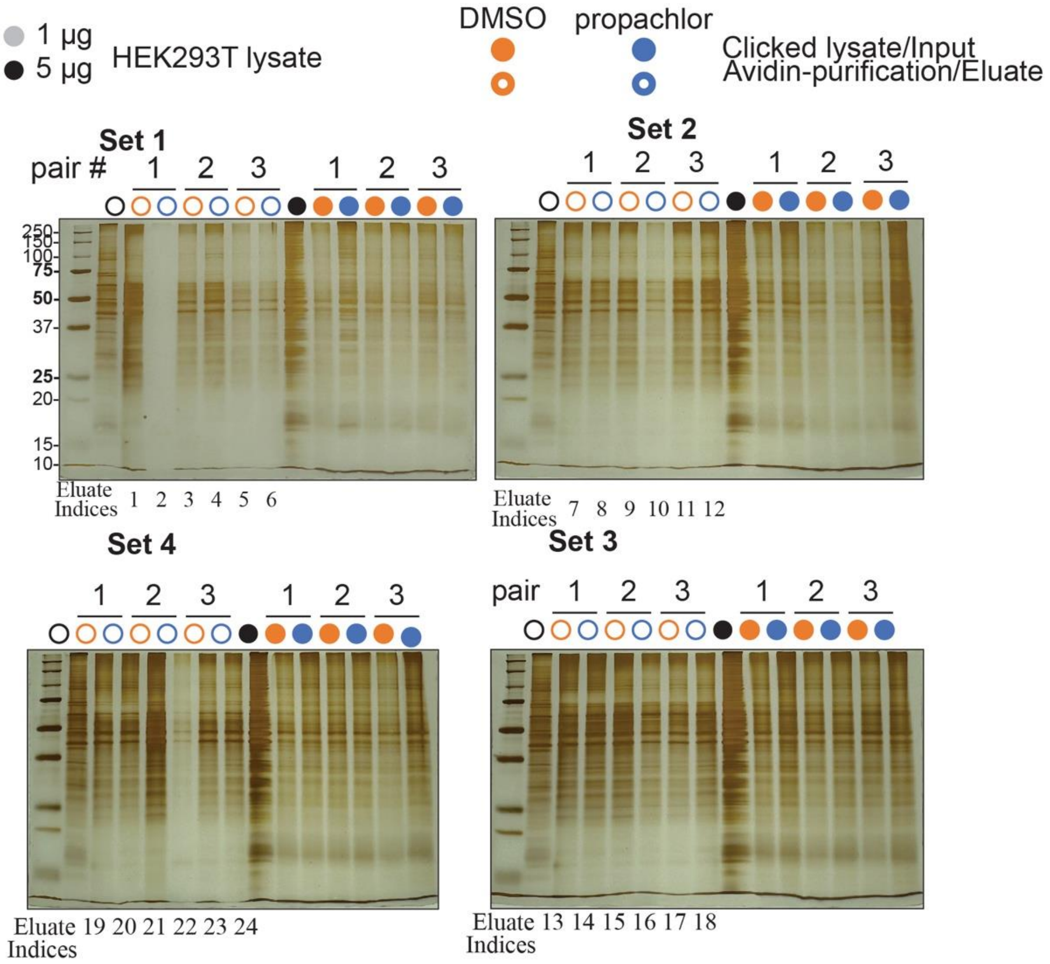

(Set, pair, ∓prop)		TMT 6-plex 126	127N	128C	129N	130C	131
Set 5		(5,1,−)	(5,1,+)	(5,2,−)	(5,2,+)	(5,3,−)	(5,3,+)
Mix & Match	1	(1,1,−)	(1,1,+)	(1,2,−)	(2,2,+)	(4,2,−)	(4,2,+)
	2	(2,1,−)	(2,1,+)	(2,2,−)	(1,2,+)	(2,3,−)	(2,3,+)
	3	(3,1,−)	(3,1,+)	(3,2,−)	(3,2,+)	(3,3,−)	(3,3,+)
	4	(4,1,−)	(4,1,+)	(1,3,−)	(1,3,+)	(4,3,−)	(4,3,+)

Eluate Indices		TMT 6-plex 126	127N	128C	129N	130C	131
Mix & Match	1	1	2	3	10	21	22
	2	7	8	9	4	11	12
	3	13	14	15	16	17	18
	4	19	20	5	6	23	24

Figure S11. Silver stains for ABPP clicked lysates and eluates. After having checked quality with set 5, we proceeded to avidin purify sets 1-4. All samples were prepared the same week, such that assignment to sets is arbitrary. Because there were some lanes with uneven amounts of total protein recovery, we assigned lanes to sets so that the lanes with lower amounts of protein (lanes 2, 10, 21, 22) were all assigned to the same TMT run. Ultimately, the normalized TMT ratios were similar across all experiments.

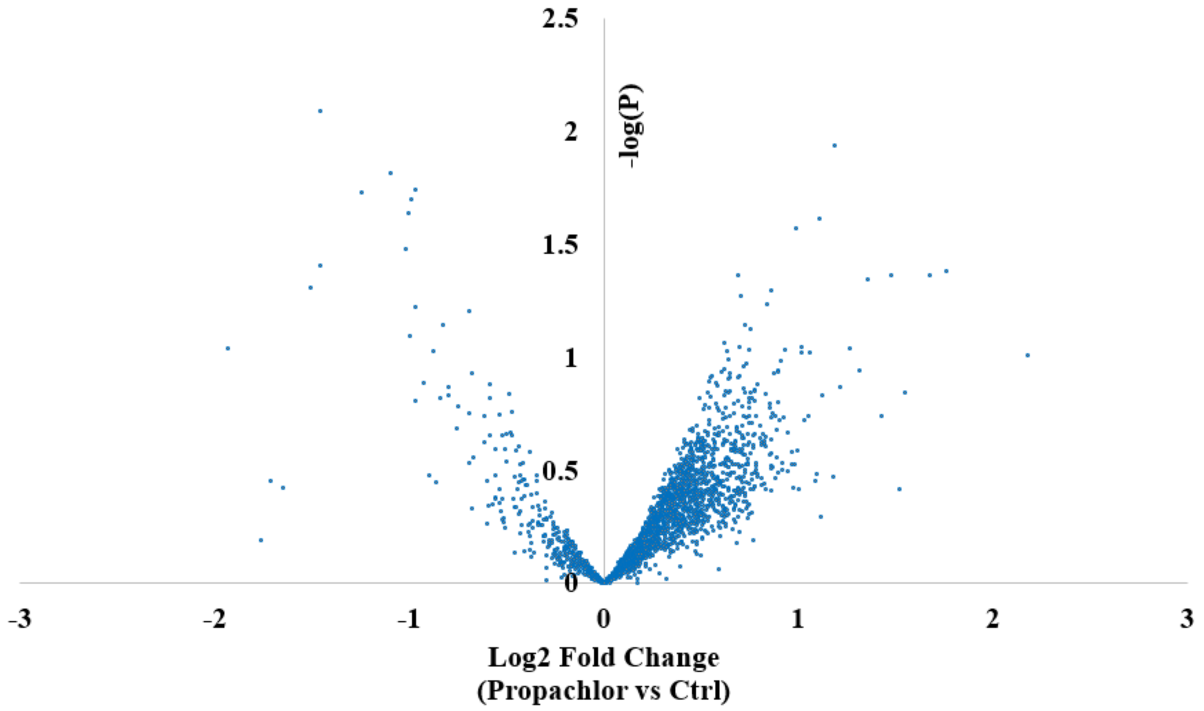

Figure S12: Differential whole cell proteomics for propachlor vs. vehicle treated HEK239T cells. 10-cm plates of HEK293T cells were treated with either 1 mM propachlor or vehicle (DMSO) for 30 min. Samples were lysed after 6 hours of recovery and aliquots were labeled with Tandem Mass Tags (TMT) and run by MuDPIT-MS. p-values were moderated with Kammers' algorithm in R[7]. No proteins were significantly changed in the total protein fraction (n = 6 biological replicates).

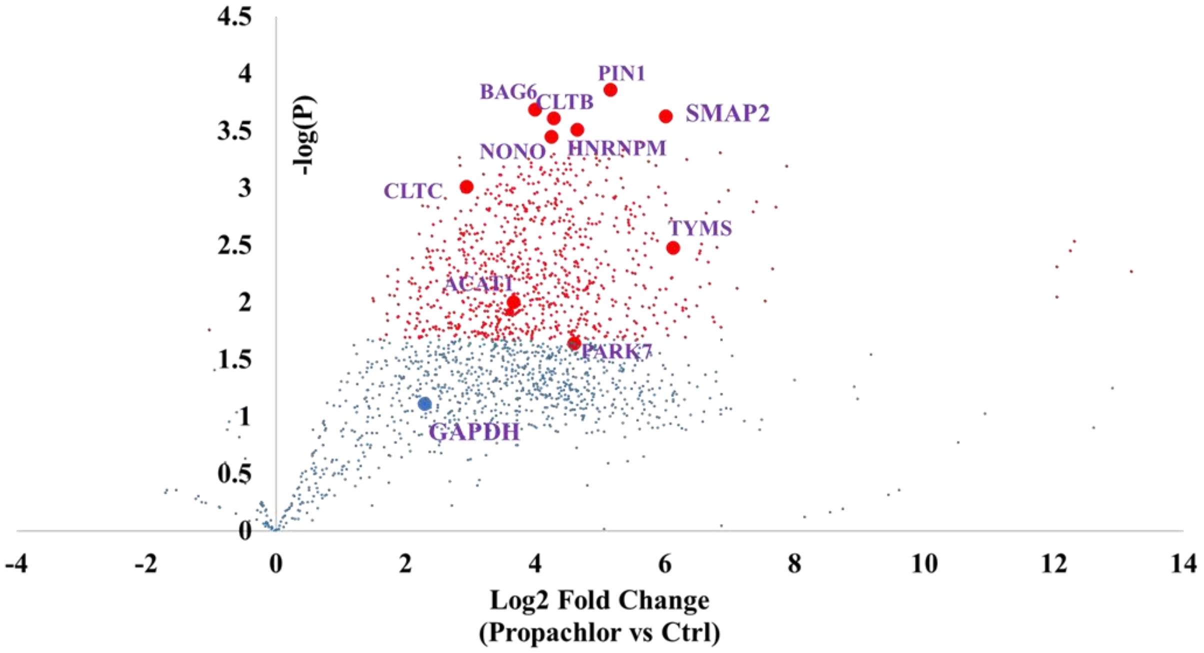

Figure S13: TMT analysis of Pellet Fractions. 10cm plates of HEK293T cells were treated with either 1 mM propachlor or vehicle (DMSO) for 30 minutes. After 6 hours of recovery and ultracentrifugation, 12 Insoluble fractions were labeled and analyzed with TMT. p-values were moderated from R calculations[4]. 826 Proteins were found to significantly aggregate according to Benjamini-Hochberg analysis including previously identified DNAJB8^{H31Q} interactors such as ACAT1, PARK7, and TYMS. The vesicular trafficking proteins[8, 9] SMAP2, GAK, CLTA, CLTB, CLTC, CLTC1, AP1B1, AP1M1, EPS15, and CLINT1 all substantially lose solubility in response to propachlor treatment, but this network only modestly increases its DNAJB8 affinity. These proteins rely on Hsp70 for clathrin disaggregation, but GAK serves as a dedicated Hsp40 co-chaperone outside of neuronal cells[10,11]. SMAP2 is involved in vesicular trafficking with Clathrin Heavy chain (CLTB)[5]. GAPDH was not among the most significant proteins aggregating into the pellet fraction (n = 6 biological replicates).

A

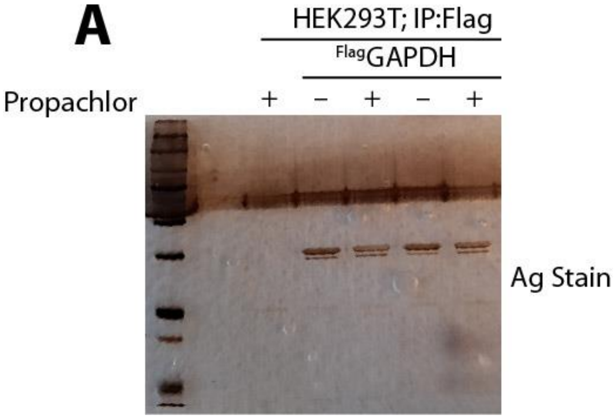

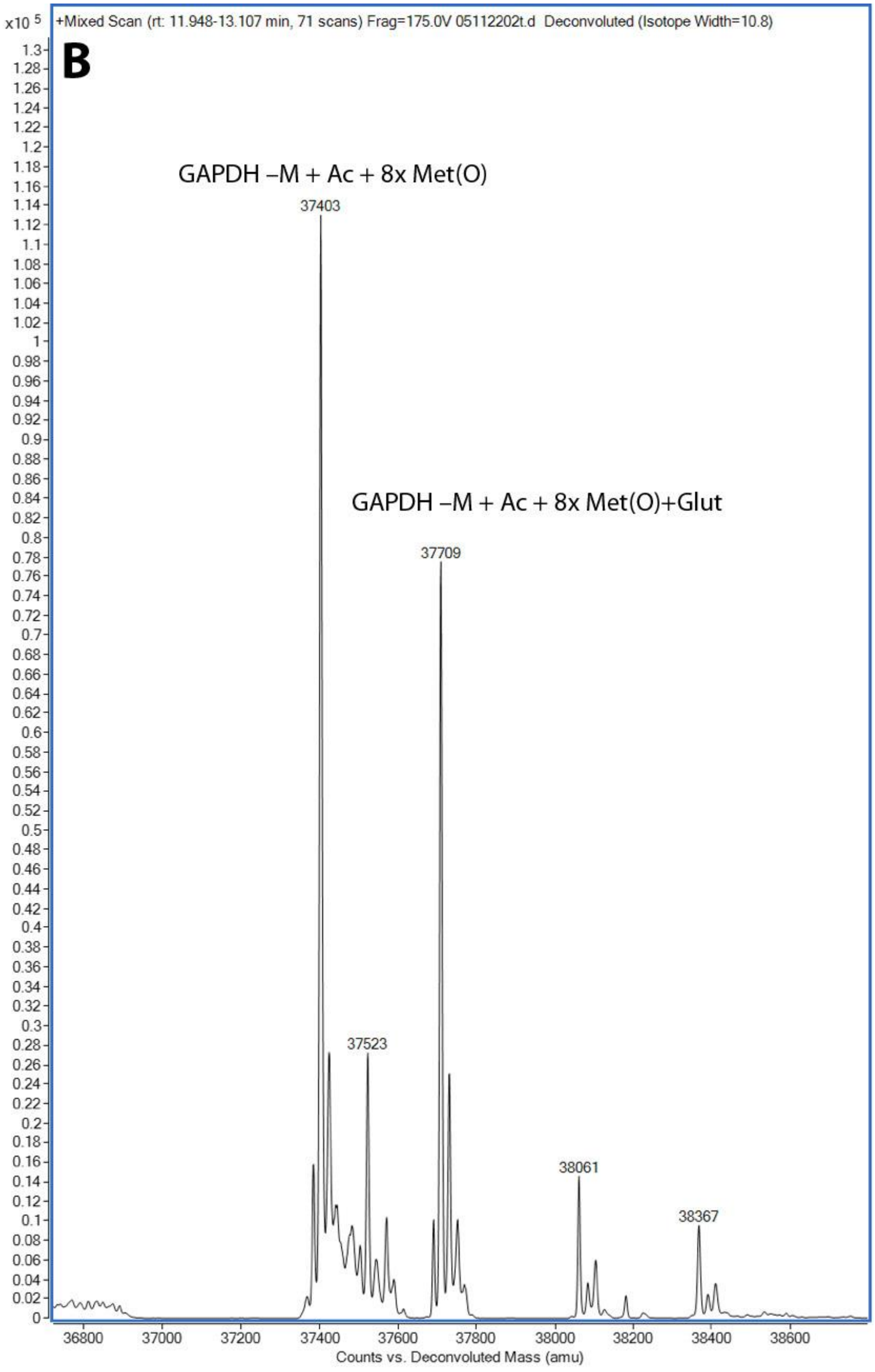

+Mixed Scan (rt: 11.948-13.107 min, 71 scans) Frag=175.0V 05112202t.d Deconvoluted (Isotope Width=10.8)
x10 5
B
GAPDH –M + Ac + 8x Met(O)
37403
GAPDH –M + Ac + 8x Met(O)+Glut
37709
37523
38061
38367
Counts vs. Deconvoluted Mass (amu)

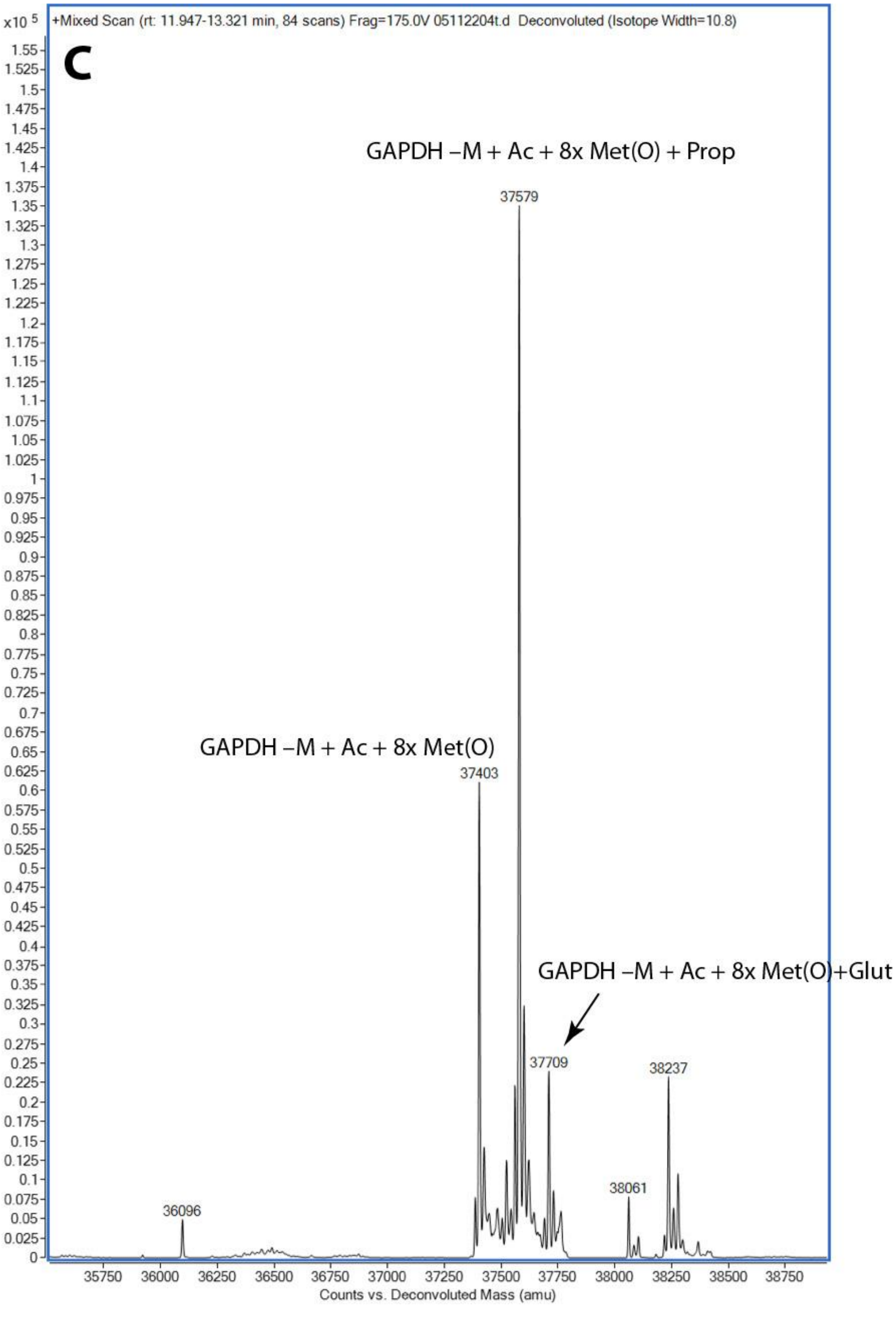

Figure S14. A. Silver stain of FlagGAPDH immunoprecipitates. **B**. Representative deconvoluted mass spectrum of FlagGAPDH treated with vehicle (DMSO). **C.** Representative deconvoluted mass spectrum of FlagGAPDH treated by propachlor.

^{Flag}GAPDH was transfected onto 10 cm of HEK293T cells by Calcium Phosphate transfection. Cells were treated with either 1 mM propachlor or vehicle (DMSO) for 30 minutes. Cells were immediately harvested and ^{Flag}GAPDH was purified by Flag immunoprecipitation. ^{Flag}GAPDH were eluted from magnetic M2 beads in 8.8 M urea. Note from the silver stain that a small amount of untagged GAPDH co-elutes; this suggests some incorporation of endogenous GAPDH into mixed heterotetramers. Samples were injected into Q-TOF LC/MS by Agilent for detection of modifications by propachlor. [M+H] is ^{Flag}GAPDH. Acetyl refers to N-terminal acetylation (+42). –M refers to aminopeptidase removal of initiator methionine. Met(O) refers to oxidation of all 8 methionines following removal of initiator methionine (+144). Glut refers to the known formation of a glutathione mixed disulfide on GAPDH (+306)[12].

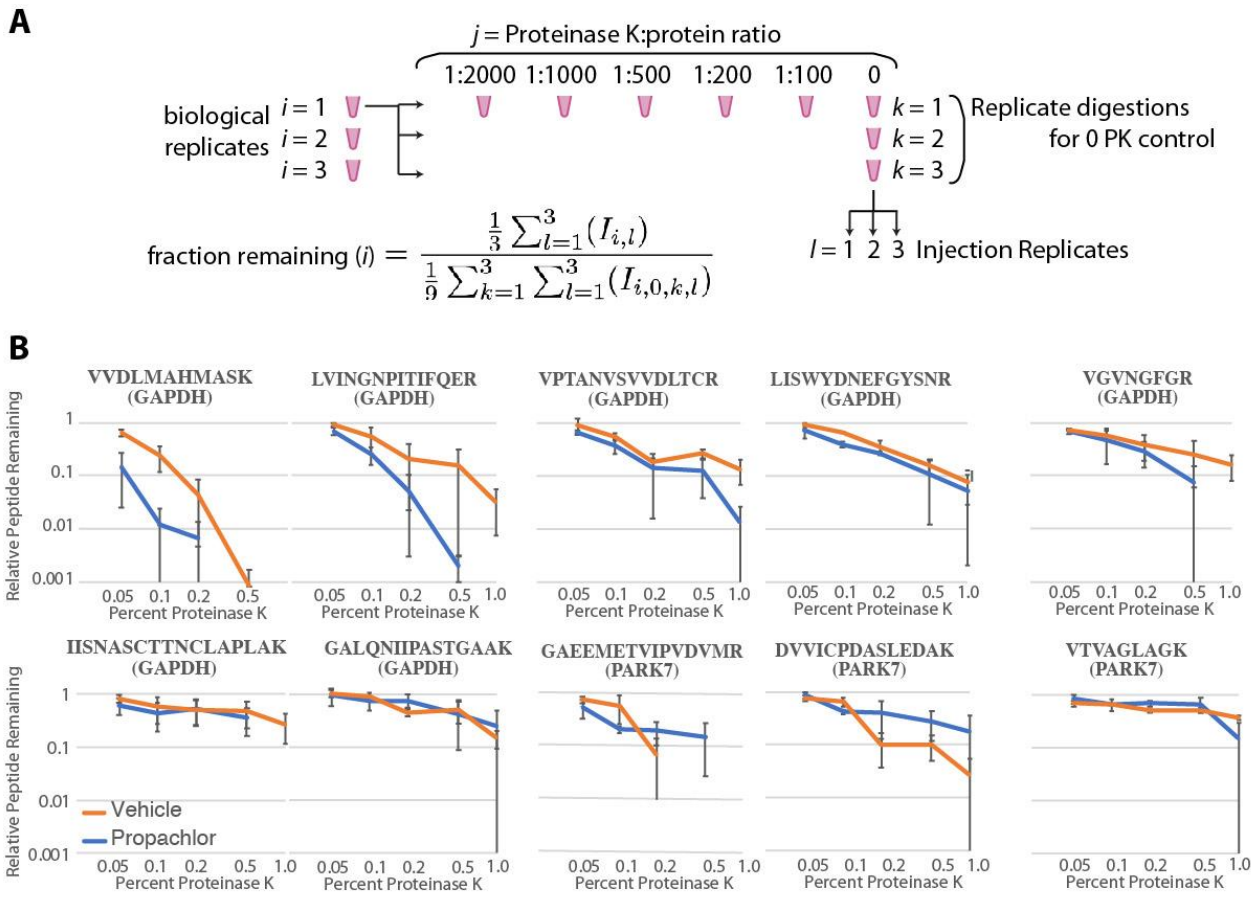

Figure S15: A) Experimental scheme for LiP experiments. Each biological replicate lysate was distributed for four different Proteinase K (PK) treatments, along with three aliquots that were not PK-treated. These samples were separately processed and digested for mass spectrometry, and each sample was analyzed with three technical replicates. **B.** Proteinase K susceptibility curves for GAPDH and PARK7 peptides. Samples from vehicle-treated cells are in orange and samples from propachlor-treated cells are in blue. (n = 3 biological replicates). LC-PRM runs were performed in technical triplicate for set A,B and averaged. Error bars represents standard error across biological replicates. Representative chromatographic traces from Skyline are presented in **Figure S18**.

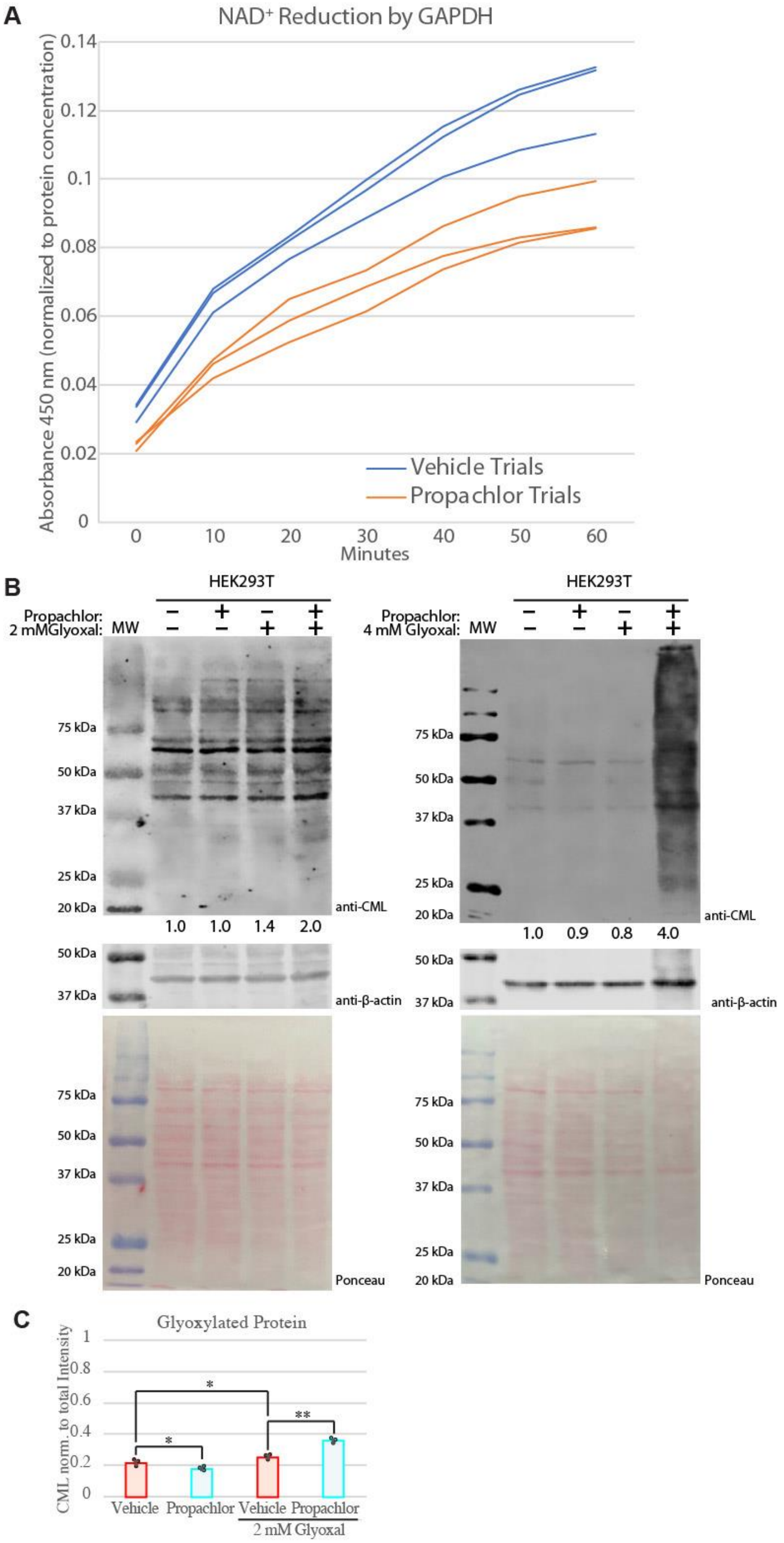
A
NAD+ Reduction by GAPDH
Absorbance 450 nm (normalized to protein concentration)
0.14
0.12
0.1
0.08
0.06
0.04
0.02
0
0 10 20 30 40 50 60
Minutes
Vehicle Trials
Propachlor Trials
B
HEK293T
Propachlor: − + − +
2 mMGlyoxal: MW − − + +
75 kDa
50 kDa
37 kDa
25 kDa
20 kDa
anti-CML
1.0 1.0 1.4 2.0
anti-β-actin
Ponceau
HEK293T
Propachlor: − + − +
4 mM Glyoxal: MW − − + +
anti-CML
1.0 0.9 0.8 4.0
anti-β-actin
Ponceau
C
Glyoxylated Protein
CML norm. to total Intensity
1
0.8
0.6
0.4
0.2
0
*
**
Vehicle Propachlor Vehicle Propachlor
2 mM Glyoxal

Figure S16. Activity assays for GAPDH and PARK7 function in propachlor treated cells. A. Kinetic traces for GAPDH activity assays, with Abs_{450} normalized to the lysate protein concentration (mg/mL) determined from Bradford assay. Each sample had two technical replicates, which were averaged together. Colorimetric measurements were taken every 10 minutes starting with initial addition of substrate. (n=3 biological replicates).**B**. Representative immunoblots, with accompanying Ponceau stains, for propachlor and glyoxal treated HEK293T cells. Western blots were blotted for CML, imaged with near-IR secondary antibodies, and the densitometry of the entire lane normalized to the total densitometry across all four samples. 30 minutes of 1 mM Propachlor before 8 additional hours of 4 mM glyoxal treatment led to more detected carboxy methyl lysine adducts (CML). **C**. Quantification of the 2 mM glyoxal experiment. Error bars represent standard deviation across three replicates. 2-way ANOVA yields $F = 55.4 > F_{crit} = 4.07$, and Tukey's HSD finds propachlor + glyoxal condition mean differences compared to all other conditions exceeds the q_{crit} for 0.01 (indicated by **). All comparisons have HSDs exceeding the $q_{0.05}$ (indicated by *).

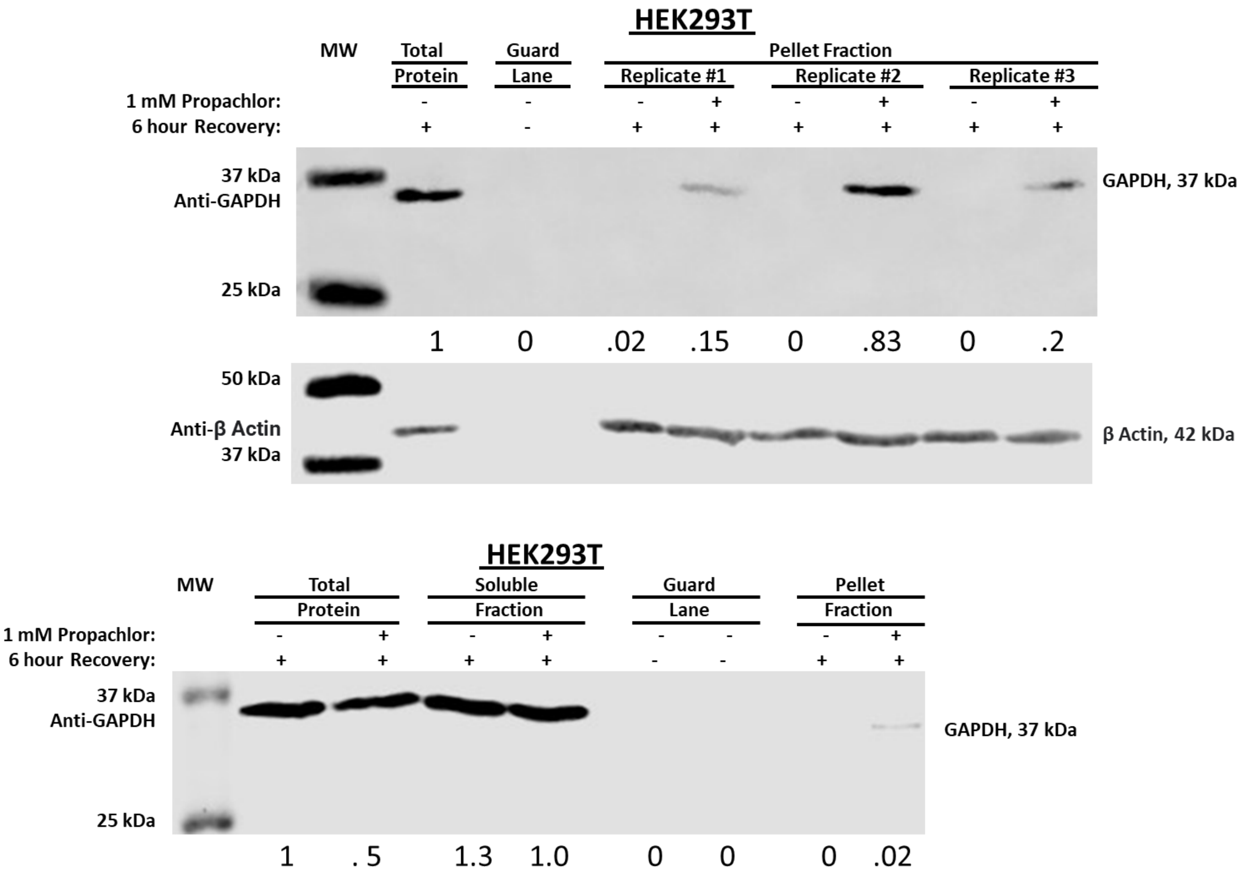

Figure S17. A. Representative Western Blot analysis of GAPDH among three biological replicates looking at 30% of the pellet fraction. 30% of each insoluble fraction from three biological replicates was loaded and ran on 10% SDS page gels. Western Blots were blotted for GAPDH and β-Actin. Predicted weights for antibodies are shown on the right. Numerical values below anti-GAPDH slice are band intensities normalized to the total fraction. GAPDH is significantly more present in Propachlor treated insoluble fractions. B. Representative Western Blot comparing 1.6 % of initial total protein, soluble fraction, and insoluble fraction in one biological replicate. 1.6% of each fraction was loaded into

each lane. Predicted weights for antibodies are shown on the right. GAPDH bands were normalized to the total protein band in untreated sample. The amount of GAPDH does not significantly decrease from total protein to soluble fraction after ultracentrifugation. Small amounts of GAPDH can be seen in the pellet fraction for propachlor-treated sample. Note that the band intensity in the soluble fraction is not noticeably affected by propachlor treatment.

Figure S18:

Peptide Sequence	Position	Mass
VVDLMAHMASK	324-334	601.307

GAPDH

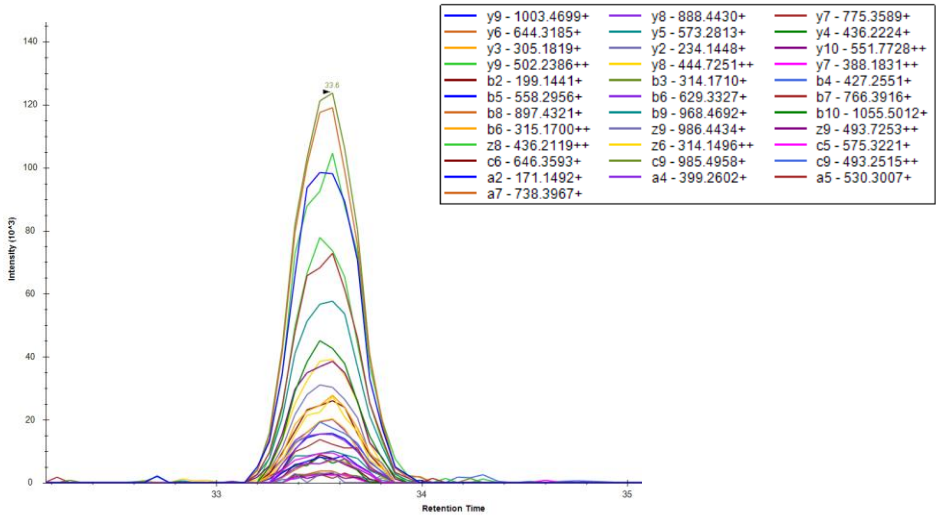

Peptide Sequence	Position	Mass
LVINGNPITIFQER	67-80	807.4541

GAPDH

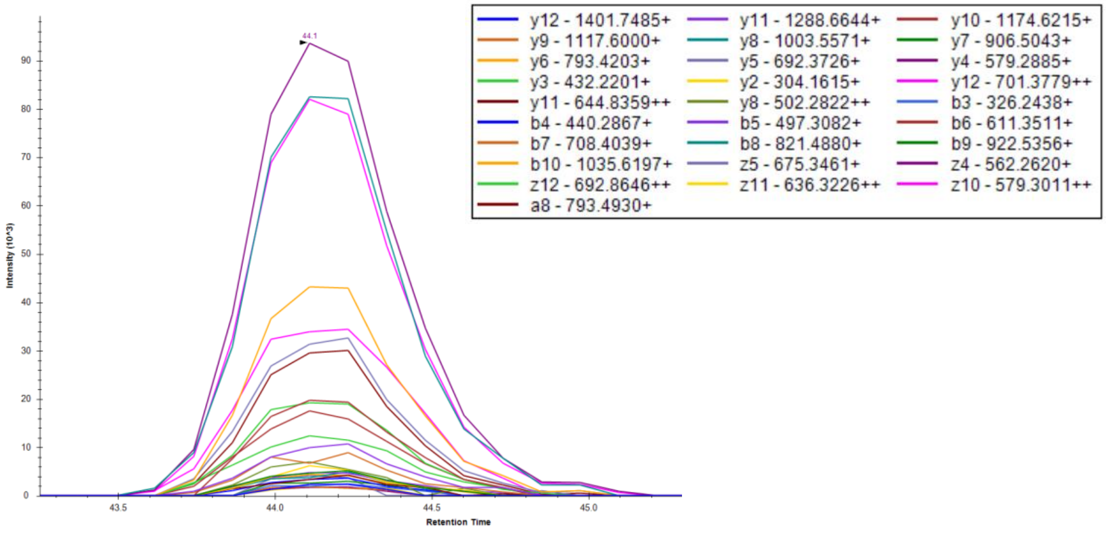

Peptide Sequence	Position	Mass
VPTANVSVVDLTCR	235-248	765.9008

GAPDH

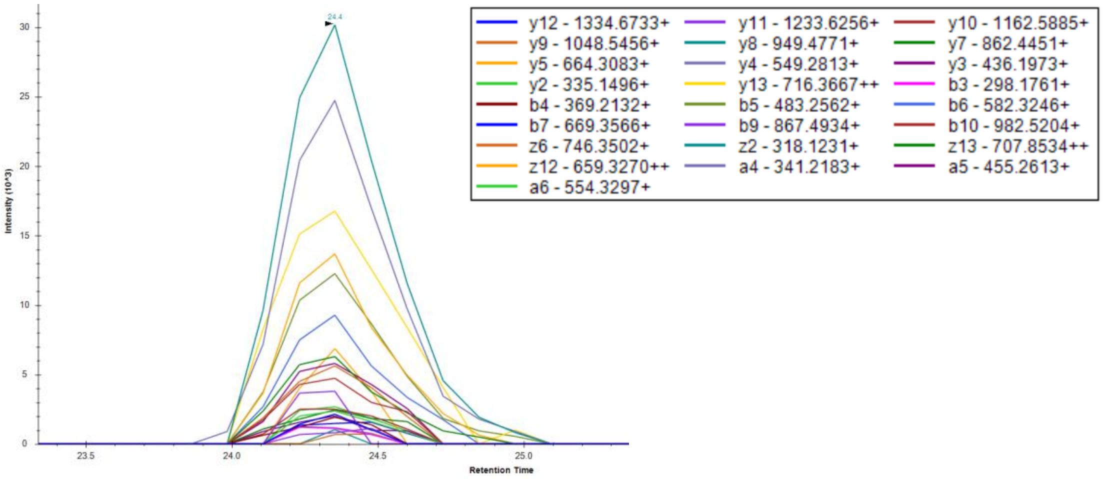

Peptide Sequence	Position	Mass
LISWYDNEFGYSNR	310-323	882.4048

GAPDH

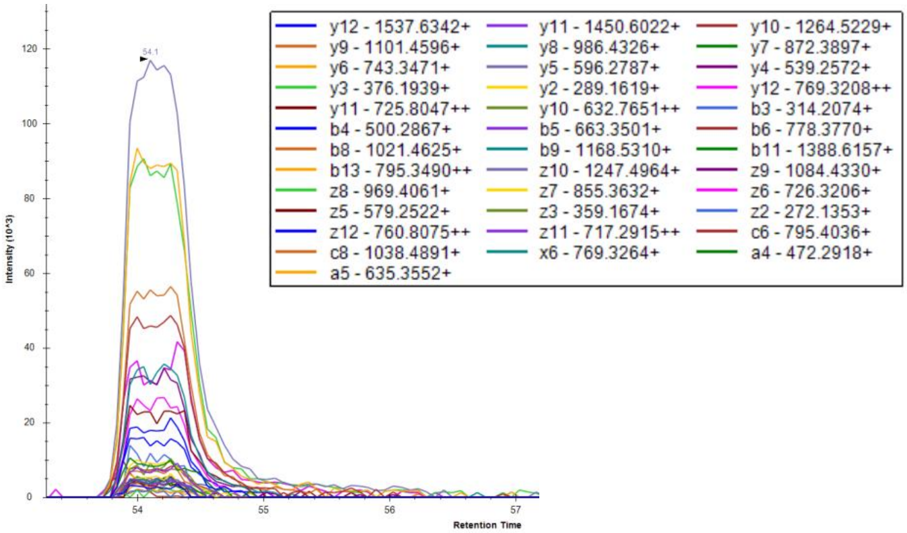

Peptide Sequence	Position	Mass
GALQNIIPASTGAAK	201-215	706.3988

GAPDH

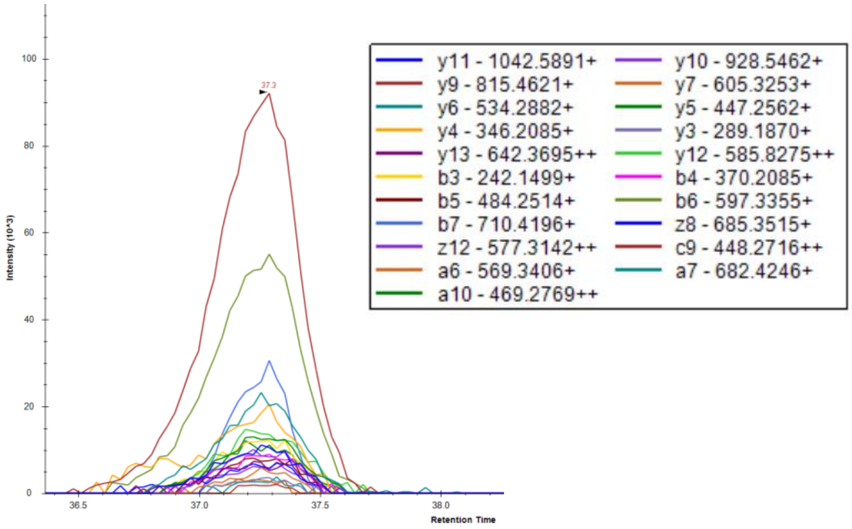

Peptide Sequence	Position	Mass
IISNASCTTNCLAPLAK	146-162	917.4635

GAPDH

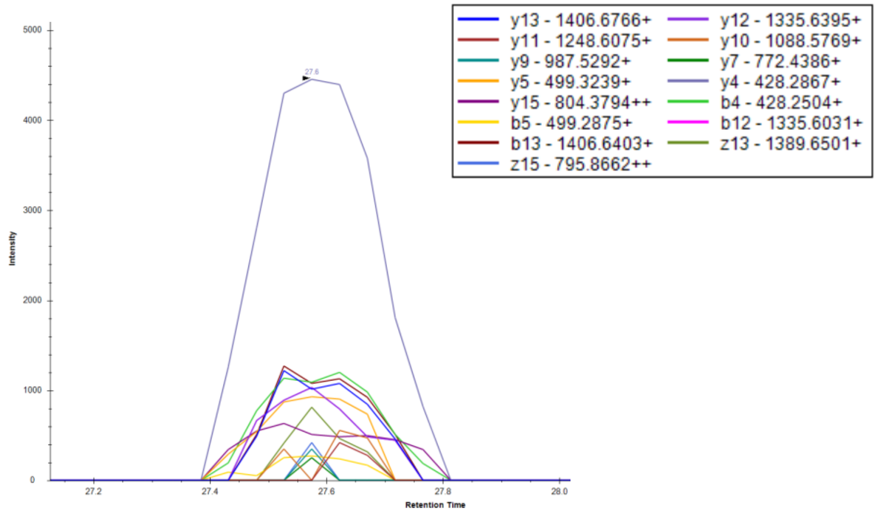

Peptide Sequence	Position	Mass
IISNASCTTNCLAPLAK	146-162	977.0065

GAPDH

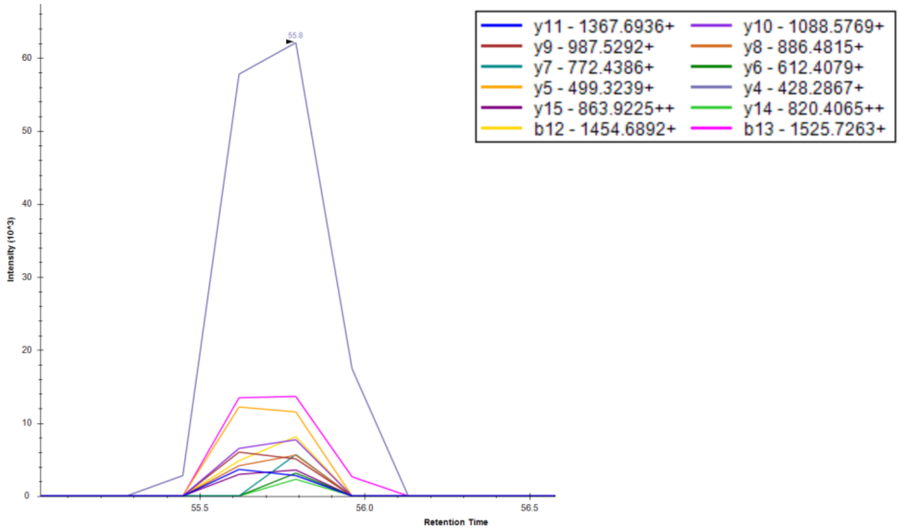

Position	Peptide Sequence	Mass
13-27	GAEEMETVIPVDVMR	838.4051

PARK7

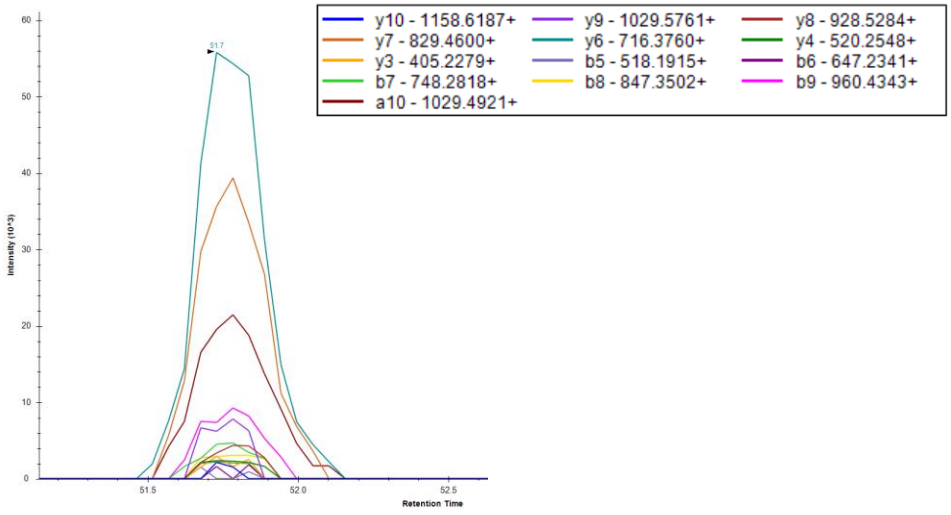

Position	Peptide Sequence	Mass
49-62	DVVICPDASLEDAK	766.369

PARK7

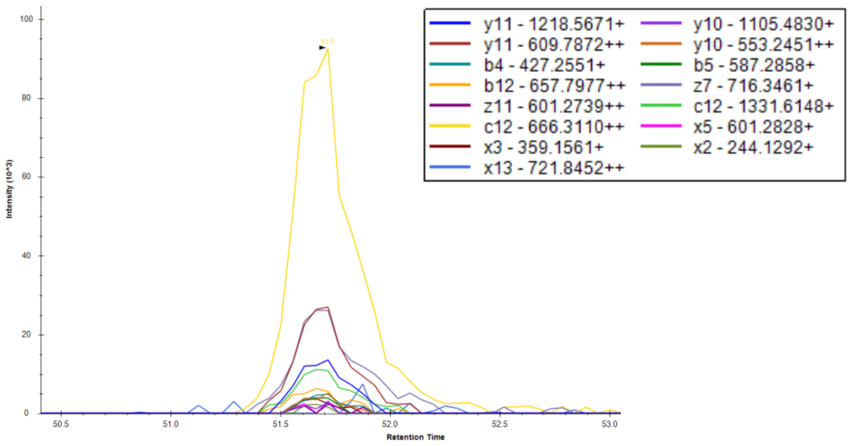

Position	Peptide Sequence	Mass
33-41	VTVAGLAGK	408.2529

PARK7

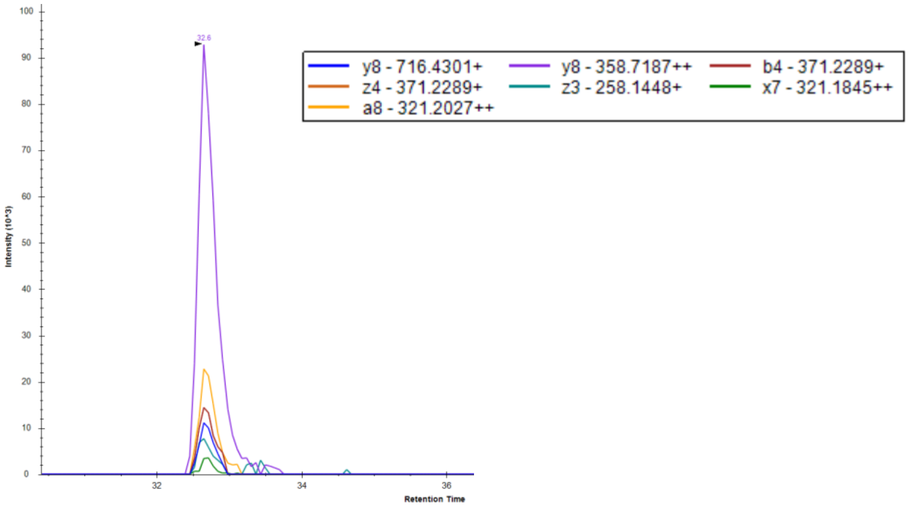

References

(1) Powers, E. T.; Gierasch, L. M. The Proteome Folding Problem and Cellular Proteostasis. *Journal of Molecular Biology* **2021**, *433* (20), 167197. https://doi.org/10.1016/j.jmb.2021.167197.
(2) Counihan, J. L.; Duckering, M.; Dalvie, E.; Ku, W.; Bateman, L. A.; Fisher, K. J.; Nomura, D. K. Chemoproteomic Profiling of Acetanilide Herbicides Reveals Their Role in Inhibiting Fatty Acid Oxidation. *ACS Chem. Biol.* **2017**, *12* (3), 635–642. https://doi.org/10.1021/acschembio.6b01001.
(3) Lambert, G. R.; Padgett, W. T.; George, M. H.; Kitchin, K. T.; Nesnow, S. Quantitative Analysis of Alachlor Protein Adducts by Gas Chromatography–Mass Spectrometry. *Analytical Biochemistry* **1999**, *268* (2), 289–296. https://doi.org/10.1006/abio.1998.3060.
(4) Liu, X.; Ling, Z.; Zhou, X.; Ahmad, F.; Zhou, Y. Comprehensive Spectroscopic Probing the Interaction and Conformation Impairment of Bovine Serum Albumin (BSA) by Herbicide Butachlor. *Journal of Photochemistry and Photobiology B: Biology* **2016**, *162*, 332–339. https://doi.org/10.1016/j.jphotobiol.2016.07.005.
(5) Quanrud, G. M.; Montoya, M. R.; Mei, L.; Awad, M. R.; Genereux, J. C. Hsp40 Affinity to Identify Proteins Destabilized by Cellular Toxicant Exposure. *Anal. Chem.* **2021**, *93* (50), 16940–16946. https://doi.org/10.1021/acs.analchem.1c04230.
(6) Anckar, J.; Sistonen, L. Regulation of HSF1 Function in the Heat Stress Response: Implications in Aging and Disease. *Annu Rev Biochem* **2011**, *80*, 1089–1115. https://doi.org/10.1146/annurev-biochem-060809-095203.
(7) Kammers, K.; Cole, R. N.; Tiengwe, C.; Ruczinski, I. Detecting Significant Changes in Protein Abundance. *EuPA Open Proteomics* **2015**, *7*, 11–19. https://doi.org/10.1016/j.euprot.2015.02.002.
(8) Kaksonen, M.; Roux, A. Mechanisms of Clathrin-Mediated Endocytosis. *Nat Rev Mol Cell Biol* **2018**, *19* (5), 313–326. https://doi.org/10.1038/nrm.2017.132.
(9) Natsume, W.; Tanabe, K.; Kon, S.; Yoshida, N.; Watanabe, T.; Torii, T.; Satake, M. SMAP2, a Novel ARF GTPase-Activating Protein, Interacts with Clathrin and Clathrin Assembly Protein and Functions on the AP-1-Positive Early Endosome/Trans-Golgi Network. *Mol Biol Cell* **2006**, *17* (6), 2592–2603. https://doi.org/10.1091/mbc.e05-10-0909.
(10) Yu, A.; Shibata, Y.; Shah, B.; Calamini, B.; Lo, D. C.; Morimoto, R. I. Protein Aggregation Can Inhibit Clathrin-Mediated Endocytosis by Chaperone Competition. *Proc. Natl. Acad. Sci. U.S.A.* **2014**, *111* (15), E1481-1490. https://doi.org/10.1073/pnas.1321811111.
(11) He, K.; Song, E.; Upadhyayula, S.; Dang, S.; Gaudin, R.; Skillern, W.; Bu, K.; Capraro, B. R.; Rapoport, I.; Kusters, I.; Ma, M.; Kirchhausen, T. Dynamics of Auxilin 1 and GAK in Clathrin-Mediated Traffic. *J Cell Biol* **2020**, *219* (3), e201908142. https://doi.org/10.1083/jcb.201908142.
(12) Barinova, K. V.; Serebryakova, M. V.; Muronetz, V. I.; Schmalhausen, E. V. S-Glutathionylation of Glyceraldehyde-3-Phosphate Dehydrogenase Induces Formation of C150-C154 Intrasubunit Disulfide Bond in the Active Site of the Enzyme. *Biochim Biophys Acta Gen Subj* **2017**, *1861* (12), 3167–3177. https://doi.org/10.1016/j.bbagen.2017.09.008.
